# Nesfatin-1 and nesfatin-1-like peptide attenuate hepatocyte lipid accumulation and nucleobindin-1 disruption modulates lipid metabolic pathways

**DOI:** 10.1038/s42003-024-06314-2

**Published:** 2024-05-27

**Authors:** Atefeh Nasri, Mateh Kowaluk, Scott B. Widenmaier, Suraj Unniappan

**Affiliations:** 1 Laboratory of Integrative Neuroendocrinology, Department of Veterinary Biomedical Sciences, Western College of Veterinary Medicine, University of Saskatchewan, Saskatoon, S7N 5B4 Saskatchewan Canada; 2 Department of Anatomy, Physiology and Pharmacology, College of Medicine, University of Saskatchewan, Saskatoon, S7N 5E5 Saskatchewan Canada

**Keywords:** Dyslipidaemias, Fat metabolism

## Abstract

Nesfatin-1 (NESF-1) has been shown to modulate lipid metabolism. We have identified a nesfatin-1-like-peptide (NLP) processed from a related precursor nucleobindin 1 (NUCB1). Here we determined if NLP, like NESF-1, regulates lipid accumulation in vitro, and tested if the disruption of nucb1 gene affects hepatic lipid metabolism genes in mice. Hepatocytes (HepG2/C3A cells) express NLP and NESF-1 and both peptides significantly reduced lipogenic enzyme mRNAs and enhanced beta-oxidation enzyme mRNAs. Lipid contents in oleic acid induced HepG2/C3A cells were attenuated by NESF-1 and NLP. The inhibitory effect on cellular lipid content was blocked by compound C, an inhibitor of AMPK. The disruption of *nucb1* gene affected lipid metabolism-related enzyme mRNAs, endogenous *nucb2* mRNA and AMPK phosphorylation. The lipid-lowering effects identified here highlights the potential of nucleobindins and peptides processed from them to address lipid disorders, and its possible benefits in metabolic disease management.

## Abstract (web summary)

Nesfatin-1 and nesfatin-1-like peptide reduced lipid content in oleic acid-induced HepG2/C3A cells, an effect that was blocked by an AMPK inhibitor compound C. This highlights the therapeutic potential for nesfatin-1 and NLP in lipid disorders.

## Introduction

Nucleobindin 1 (NUCB1) is a calcium- and DNA-binding, 55 kD multi-domain protein. This protein was reported in cellular Golgi apparatus^1^ and nucleus^2^ and was found to be secreted from cells^3^. NUCB1 is highly conserved in mammals and non-mammals^4^. Another member of nucleobindin family was named NUCB2 due to high similarity to NUCB1. Nesfatin-1 (NESF-1) (NEFA/nucleobindin 2 encoded satiety and fat influencing protein), an 82 amino acid peptide processed from the N-terminal region of NUCB2 was reported in 2006^5^. This peptide has received considerable attention due to scientific findings implicating its role in the regulation of food^5^ and water^6^ intake, lipid^7^ and glucose^8^ metabolism, insulinotropic effect^9^, reproduction^10^, and sleep^11^. NUCB2 is expressed in hypothalamus^5,12^, pituitary^12^, pancreas^8,13^, gastrointestinal tract^13,14^, and testis^13^. The presence of NUCB2 (precursor) and NESF-1 (processed peptide) in central and peripheral endocrine tissues suggests pleiotropic effects. NESF-1 has also been implicated in the regulation of lipid metabolism in different tissues, including white adipose tissue (WAT) and liver.

Chronic central infusion of NESF-1 reduced bodyweight gain, and subcutaneous and visceral fat mass of Wistar rats^5^. NESF-1 reduced lipid accumulation in 3T3-L1 mouse preadipocytes and inhibited the expression peroxisome proliferator-activated receptor (*Ppar) γ*, fatty acid binding protein *(Fabp) 4* and *Cfd* (adipsin) mRNAs in differentiated adipocytes^15^. On the other hand, a clear inhibition of cell proliferation and an increase in adipocyte differentiation were reported in *Nucb2* knockdown group of 3T3-L1 preadipocytes^16^. Moreover, peripheral infusion of NESF-1 attenuated lipid accumulation in hepatocytes through AMP-activated protein kinase (AMPK)-mediated pathway in diet-induced obese mice^7^. A significant reduction in serum NESF-1 in patients with non-alcoholic fatty liver (NAFLD) disease was reported^17^. These observations indicate a potential role for NESF-1 in the regulation of lipid metabolism.

Very recently, it has been proposed that NUCB1 has a NESF-1-like peptide (NLP) sequence, which is putatively processed by prohormone convertases (PCSKs). *Nucb1* and *Nucb*2 shared 60% sequence homology in the mouse genome^18^. It was reported that the bioactive core of murine NLP and NESF-1 shares 76% amino acid sequence homology^19^. The presence of NLP in similar anatomical locations as NESF-1, including the pancreas, pituitary, gonads, and gut was reported^19,20^. Similarly, NLP was shown to suppress food intake and modulate the expression of appetite-regulatory hormones in goldfish^20^ and rats. Based on the similarities on its sequence, tissue distribution, and biological actions of NLP and NESF-1, we hypothesized that NLP too elicit NESF-1 effects in decreasing lipid accumulation in hepatocytes. We tested this hypothesis using human hepatocytes (HepG2/C3A cells) and genetically modified mice.

## Results

### HepG2/C3A cells express NUCB1 and NUCB2

**Fig. 1:**
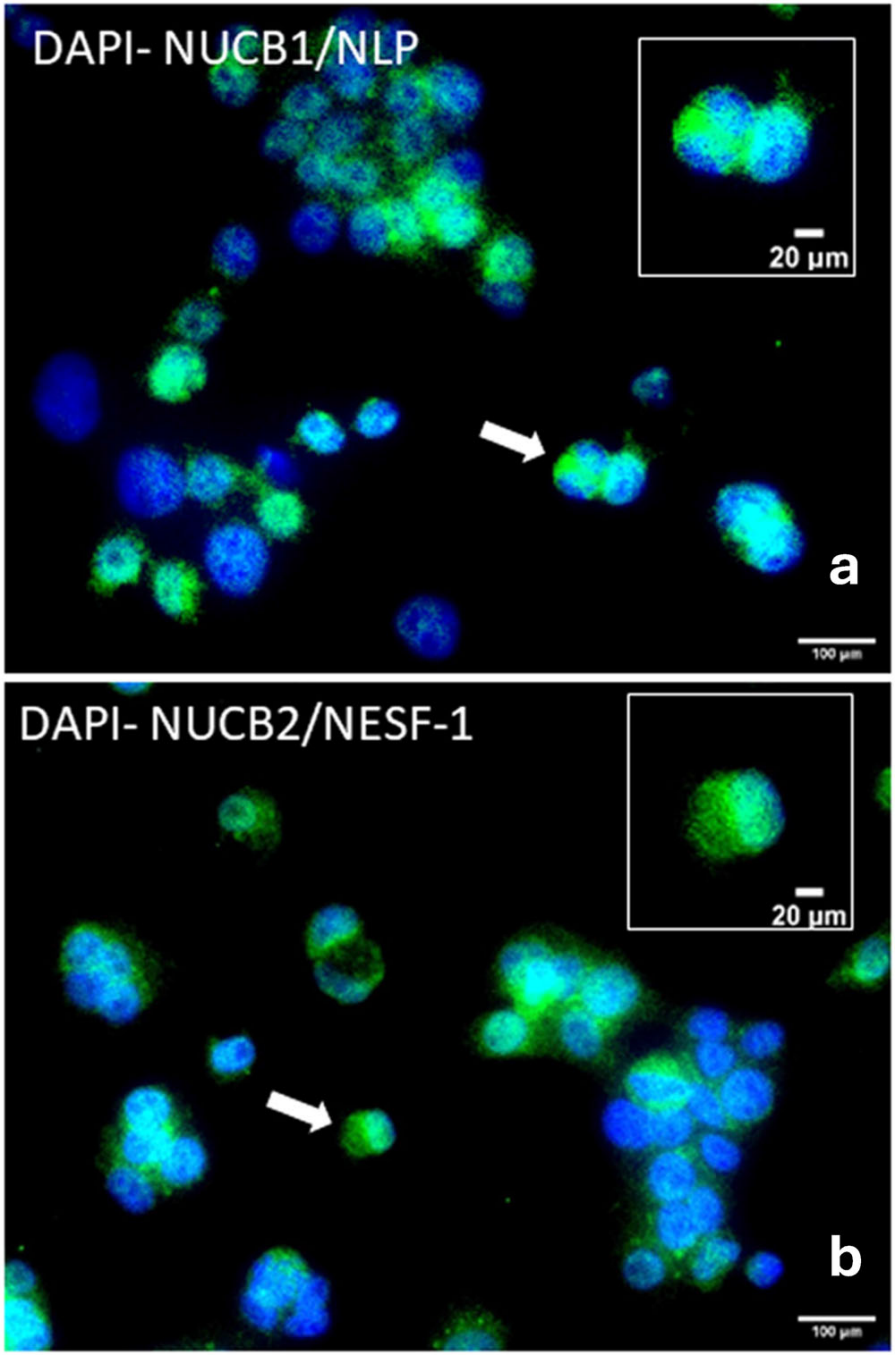
HepG2/C3A cells express NUCB1/NLP and NUCB2/NESF-1. HepG2/C3A cells express NUCB1/NLP (**a**) and NUCB2/NESF-1 (**b**) in green color. DAPI stained DNA in blue color. The arrows point to magnified hepatocytes shown in insets. Scale bar = 100 µm, or 20 µm (inset images). DAPI stained DNA in blue color. Primary antibodies used are: rabbit anti-mouse NUCB1 (1:250, custom synthesized, cat no. 1312-PAC-02, Pacific Immunology, USA), rabbit anti-mouse NUCB2 (1:250, RRID: AB_2891124, cat no. 1312-PAC-01, Pacific Immunology, USA). Secondary antibodies used are: goat anti-rabbit Alexa Fluor® 488 (1:500, RRID: AB_2630356, cat no. ab150077, ABCAM, UK).

To determine whether hepatocytes express NUCBs, localization of NUCB1 and NUCB2 in HepG2/C3A cells was carried out using immunocytochemistry (ICC) based on our previously reported protocols^21^. HepG2/C3A cells are immunoreactive for NUCB1/NLP (green, Fig. 1a) and NUCB2/NESF-1 (green, Fig. 1b). DAPI stained cell DNA in blue color. NUCB1/NLP-like immunoreactivity was mainly observed in the nucleolus. In contrast, NUCB2/NESF-1 like immunoreactivity was mainly observed in the cytoplasm of cells and was also detected in the nucleus of some cells. No immunoreactivity was observed for the negative control group, which was incubated with only the secondary antibody (Supplementary Fig. 1).

### NLP and NESF-1 affected the expression of enzymes involved in hepatic lipid synthesis in untreated and oleic acid (OA)-induced HepG2/C3A cells

**Fig. 2:**
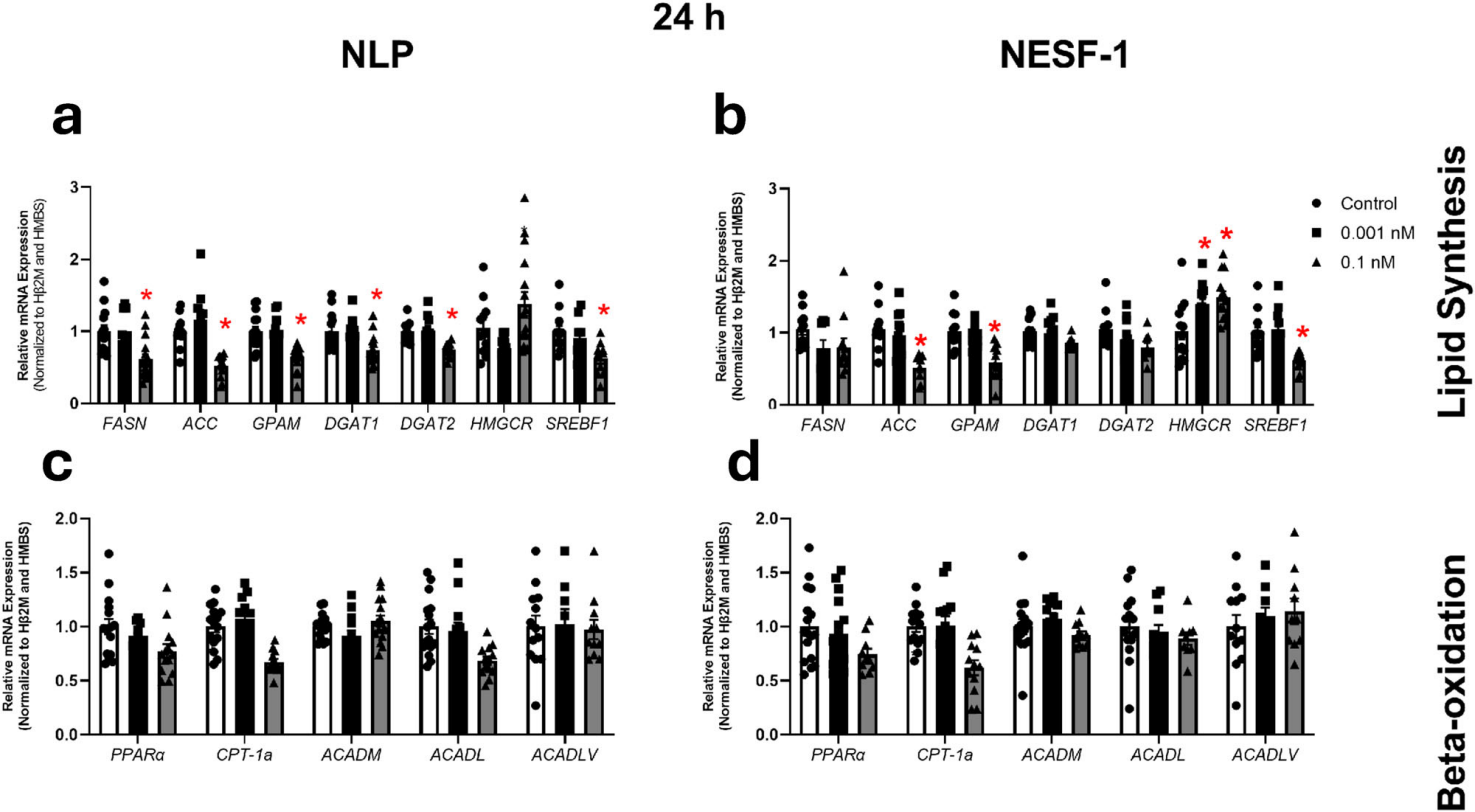
NESF-1 and NLP modulate hepatic lipogenic and beta-oxidation enzyme mRNAs. NLP and NESF-1 (0.001 or 0.1 nM) affected the expression of hepatic lipogenic (**a**, **b**) and beta-oxidation (**c**, **d**) enzyme mRNAs in HepG2/C3A cells at 24 h post incubation. All data are represented as mean ± SEM. Results presented are pooled from three independent studies with at least triplicates for each treatment within each study. Asterisks indicate significant differences between experimental group (each concentration) and corresponding control (untreated cells) for mRNA quantified. Significance was set at *P* < 0.05. mRNAs measured are: acetyl-CoA carboxylase (*ACC)*, glycerol-3-phosphate acyltransferase *(GPAM)*, sterol regulatory-element binding factor (*SREBF)-1*, carnitine palmitoyl transferase (*CPT)-1α*, 3-hydroxy-3-methylglutaryl-coA reductase (*HMGCR)*, fatty acid synthase (*FASN)*, diacyl glycerol acyltransferase (*DGAT) 1*, *DGAT2* and long/very long-chain acyl coA dehydrogenase *(ACADL; ACADLV),* peroxisome proliferator-activated receptor (*Ppar) γ,* acyl CoA dehydrogenase medium chain (ACADM).

The possible regulatory effects of NLP and NESF-1 on the abundance of mRNAs for enzymes involved in hepatic lipid synthesis and beta-oxidation were studied. As shown in Fig. 2a–d, NLP, and NESF-1 at 0.1 nM decreased lipogenic acetyl-CoA carboxylase (*ACC)*, glycerol-3-phosphate acyltransferase *(GPAM)*, sterol regulatory-element binding factor (*SREBF)-1* and carnitine palmitoyl transferase (*CPT)-1α* and increased 3-hydroxy-3-methylglutaryl-coA reductase (*HMGCR)* in untreated HepG2/C3A cells at 24 h post incubation. NLP also decreased the expression of fatty acid synthase (*FASN)*, diacyl glycerol acyltransferase (*DGAT) 1*, *DGAT2* and long-chain acyl coA dehydrogenase *(ACADL)* at the same time point.

Specifically, NLP and NESF-1 at 0.1 nM decreased lipogenic *ACC*, *GPAM*, and *SREBF-1,* but increased *HMGCR* mRNA at 24 h post incubation (Fig. 2a, b). Both peptides decreased *CPT-1α* mRNA expression in the same experiment (Fig. 2c, d). Moreover, NLP decreased the expression of *FASN*, *DGAT1*, *DGAT2*, and *ACADL* mRNA in HepG2/C3A cells at 24 h post incubation (Fig. 2a, c).

**Fig. 3:**
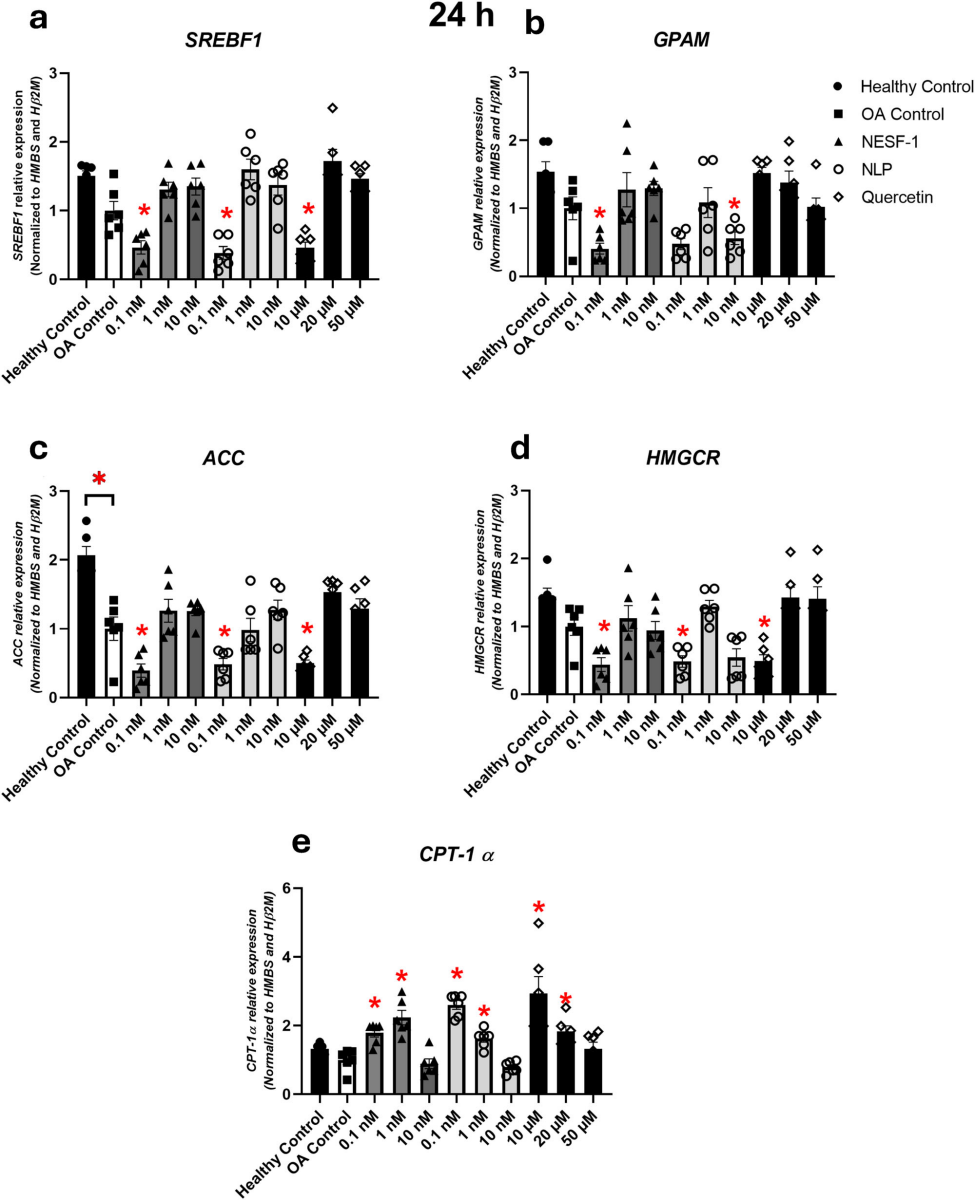
NESF-1 and NLP affected lipid metabolism enzyme mRNAs in oleic acid-treated cells. NLP and NESF-1 (0.1–10 nM) and quercetin (10 µM) affected the expression of hepatic lipogenic (**a**–**d**) and beta-oxidation (**e**) enzymes at mRNA level in OA-induced HepG2/C3A cells at 24 h post incubation. All groups were pretreated with OA except untreated controls. All data are represented as mean ± SEM. Results presented are pooled from three independent studies with at least triplicates for each treatment. Asterisks show the significant difference between the experimental group (each concentration) and the corresponding OA control for each gene. Significance was set at *P* < 0.05.

NLP and NESF-1 at 0.1 nM decreased the expression of lipogenic *ACC*, *GPAM*, *SREBF-1,* and *HMGCR*, while increasing *CPT-1α* (beta-oxidation) at 24 h post incubation (Fig. 3a–d). Positive control, quercetin, at 10 µM decreased the expression of lipogenic *ACC*, *SREBF-1* and *HMGCR* but did not show any effect on *GPAM*. Similar to NESF-1 and NLP, quercetin increased the *CPT-1α* mRNA level at 24 h post incubation.

### NLP and NESF-1 decreased Oil Red O (ORO) staining area in OA-induced HepG2/C3A cells

**Fig. 4:**
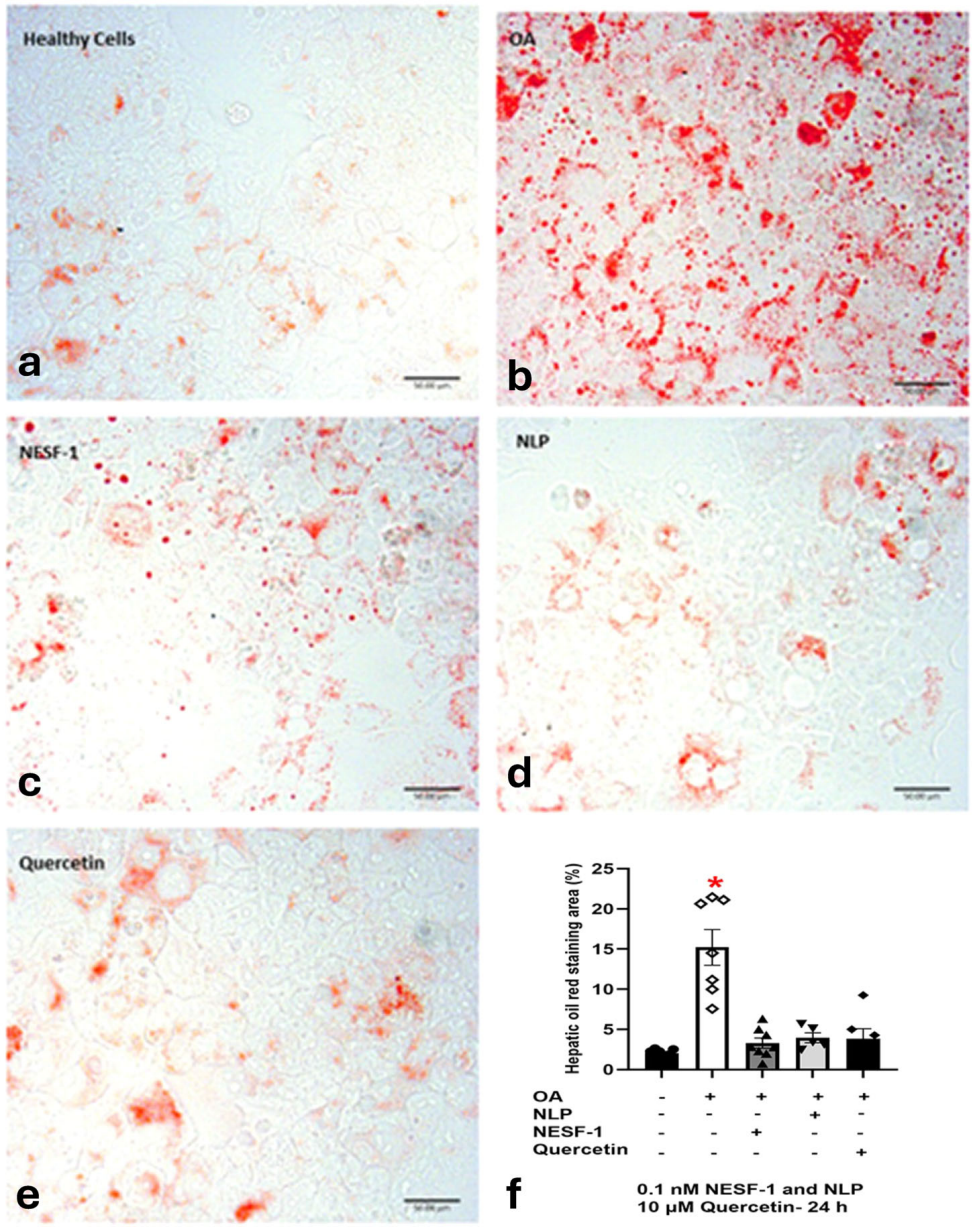
Nesfatin-1 and NLP reduced lipid content staining in oleic acid-treated cells. These pictures show Oil Red O (ORO) staining in HepG2/C3A cells (**a**). Scale bar = 50 µm. All groups were pretreated with oleic acid (OA; **b**) except untreated controls. NLP (**d**; 0.1 nM) and NESF-1 (**c**; 0.1 nM), and quercetin (**e**; 10 µM) decreased ORO lipid staining area in OA-induced HepG2/C3A cells at 24 h post incubation. HepG2/C3A cells were fixed with 4% paraformaldehyde and then stained with ORO. The ORO staining area (**f**) within cells was measured by ImageJ software (5–7 images/group). All data are represented as mean ± SEM. Asterisks show the statistically significant difference between each group and untreated controls. Significance was set at *P* < 0.05.

In order to determine whether lipid accumulation in HepG2 cells (Fig. 4a) is modulated by nesfatin-1 or NLP, we treated HepG2/C3A cells with oleic acid (Fig. 4b) and stained the cells with the lipid stain Oil Red O. NLP (Fig. 4d) and NESF-1 (Fig. 4c) decreased the ORO lipid staining area by approximately 11% when compared to OA control group (Fig. 4b) as assessed by lipid staining area (Fig. 4f) of 5–7 images per group using ImageJ software. Quercetin (positive control; Fig. 4e) also caused a similar decrease in the lipid staining area in OA-induced HepG2/C3A cells (Fig. 4f). The effect of quercetin was not statistically different from the effects of NESF-1 and NLP.

**Fig. 5:**
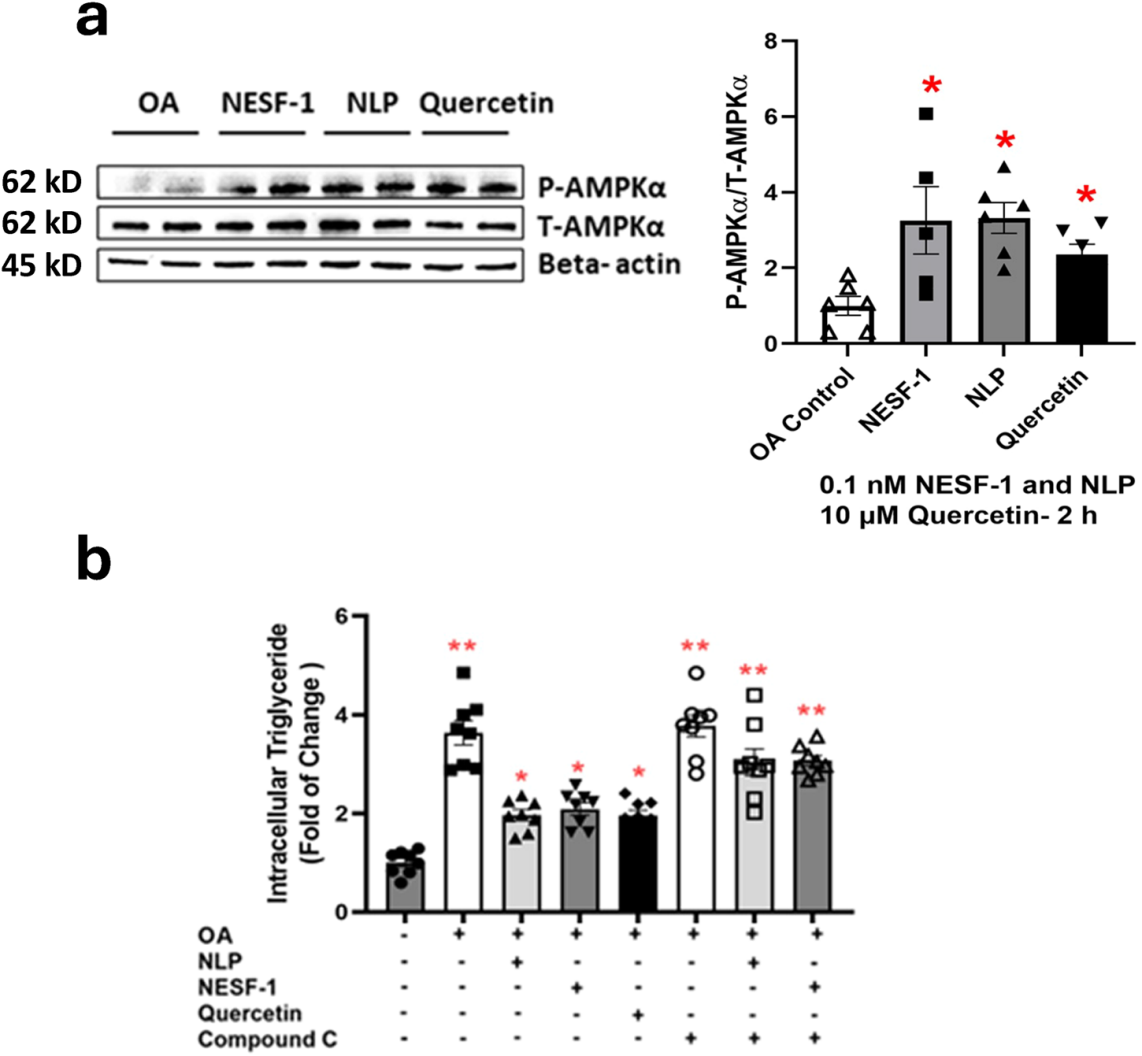
Nesfatin-1 and NLP increased AMPK phosphorylation in oleic acid-treated cells. NLP and NESF-1 (0.1 nM) and quercetin (10 µM) increased the phosphorylation of AMPK in OA-induced HepG2/C3A cells after 2 h post incubation (**a**). Primary antibodies: polyclonal rabbit anti-phospho (Thr172)-AMPKα antibody (1:1000, RRID: AB_330330, cat no. 2531 S, Cell Signalling, USA), polyclonal rabbit anti-AMPKα antibody (1:1000, RRID: AB_330331, cat no. 2532 S, Cell Signalling, USA). The secondary antibodies: goat anti-rabbit IgG (H + L)-HRP conjugate antibody (1:5000, RRID: AB_11125142, cat no. 170-6515, Bio-Rad, USA) and goat anti-mouse IgG (H + L)-HRP conjugate antibody (1: 5000, RRID: AB_11125547, cat no. 170-6516, Bio-Rad, USA). Studying the NLP and NESF-1 mechanism of action on HepG2 to decrease lipid accumulation in HepG2 cells (**b**), HepG2 cells were treated with compound c (10 µM) for 1 h and then NLP/NESF-1/Quercetin were added to media for 24 h. Results for both parts presented are pooled from three independent studies with duplicate for each treatment. All groups were pretreated with OA except untreated controls (**a**, **b**). All data are presented as mean ± SEM. Asterisk shows the significant difference between each group and OA control (**a**, **b**) while two asterisks show the significant difference between each group and untreated controls (**b**). Significance was set at *P* < 0.05.

AMPK has been suggested as an involving intracellular signaling molecule for treating several chronic diseases, such as type 2 diabetes and obesity. AMPK is a serine/threonine protein kinase that possesses a fundamental role in the regulation of cellular energy homeostasis in mammalian cells. This molecule is activated upon depletion of cellular energy^22^. Then, activated AMPK induces ATP generation pathways such as glycolysis, fatty acid oxidation and lipolysis while inhibiting anabolic pathways, including lipid and protein synthesis. Polyphenolic compounds, for example, quercetin were shown to attenuate lipid accumulation in HepG2/C3A cells through the AMPK-mediated pathway^23^. Moreover, NESF-1 was reported to increase phosphorylated (P)-AMPKα /Total(T)-AMPKα in the primary culture of mouse hepatocytes^7^. Since NLP and NESF-1 share identical amino acid sequences, especially within the putative bioactive core across vertebrates, we hypothesized that NLP and NESF-1 might act through AMPK pathway to regulate hepatic lipid synthesis and oxidation. To determine the cell signaling mediators, HepG2/C3A cells were incubated with fresh media (control group) or NLP/NESF-1/quercetin (experimental groups) at effective doses for 2 h, and then P-AMPKα/T-AMPKα ratio was assessed in cell lysate. As shown in Fig. 5a, 0.1 nM of NLP and NESF-1 significantly increased P-AMPKα/T-AMPKα ratio at 2 h post incubation. Quercetin exerted the same effects on P-AMPKα/T-AMPKα ratio. The inhibition of AMPK by compound C resulted in a significant increment of triglyceride (TG) content in OA-induced HepG2/C3A cells (Fig. 5b). Furthermore, NLP and NESF-1 attenuated the cellular TG, which was 1.8-fold less than the OA control. Quercetin exerted similar effects on cellular TG. The cellular TG levels in OA control was 3.6-fold higher than what was found in untreated HepG2/C3A cells.

### NLP and NESF-1 affected *NUCB* mRNAs and NUCB proteins in OA-induced HepG2/C3A cells

**Fig. 6:**
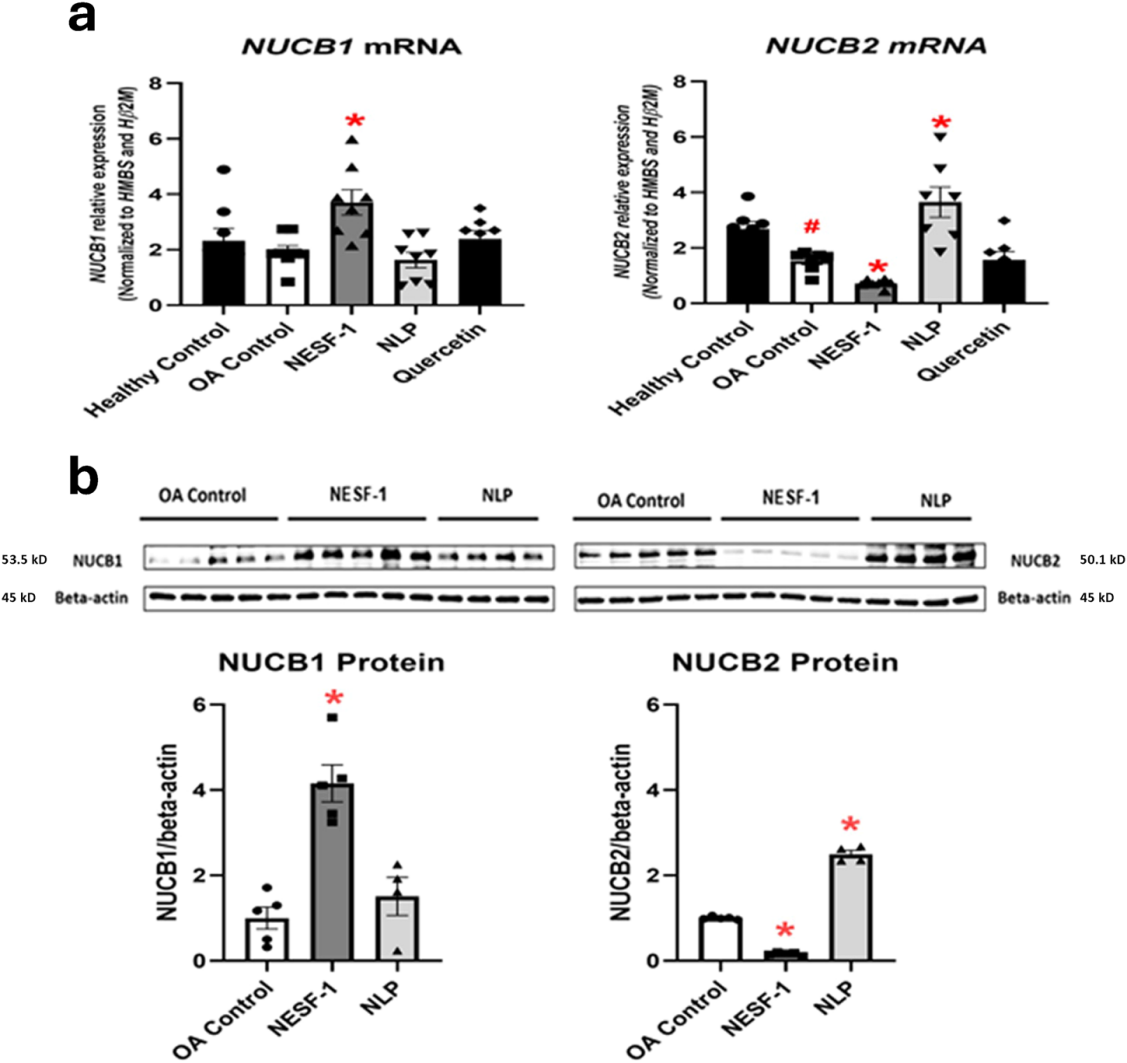
Nesfatin-1 and NLP influenced *NUCB* mRNAs and NUCB protein in oleic acid-treated cells. NLP and NESF-1 (0.1 nM) affected *NUCB* mRNA (**a**) and NUCB protein (**b**) in OA-induced HepG2/C3A cells at 24 h post incubation. All groups were pretreated with oleic acid (OA) except untreated control. Results presented for *NUCB* mRNA (**a**) are pooled from three independent studies with at least triplicates for each treatment. Results presented for NUCB protein (**b**) were pooled from two independent studies with duplicates or triplicates for each treatment (*n* = 4–5 samples in total/group). All data are represented as mean ± SEM. Asterisks show the difference between the experimental group and the OA control. Significance was set at *P* < 0.05.

The addition of nesfatin-1 or NLP could influence the abundance of endogenous forms of these peptides produced within HepG2 cells and this was tested. NESF-1 upregulated *NUCB1* mRNA while downregulated *NUCB2* mRNA. In contrast, NLP had no effects on *NUCB1* mRNA, but upregulated *NUCB2* mRNA in OA-induced cells. The endogenous level of *NUCB2* mRNA was decreased in OA-induced cells compared to untreated cells (Fig. 6a). Similarly, NESF-1 enhanced NUCB1 protein level while decreasing NUCB2 protein. Moreover, NLP enhanced NUCB2 protein level in OA-induced HepG2/C3A cells (Fig. 6b).

### The genetic disruption of *Nucb1* gene affected lipid pathways-related enzymes and *Nucb* mRNAs in mice liver in a sex- and diet-specific manner

**Fig. 7:**
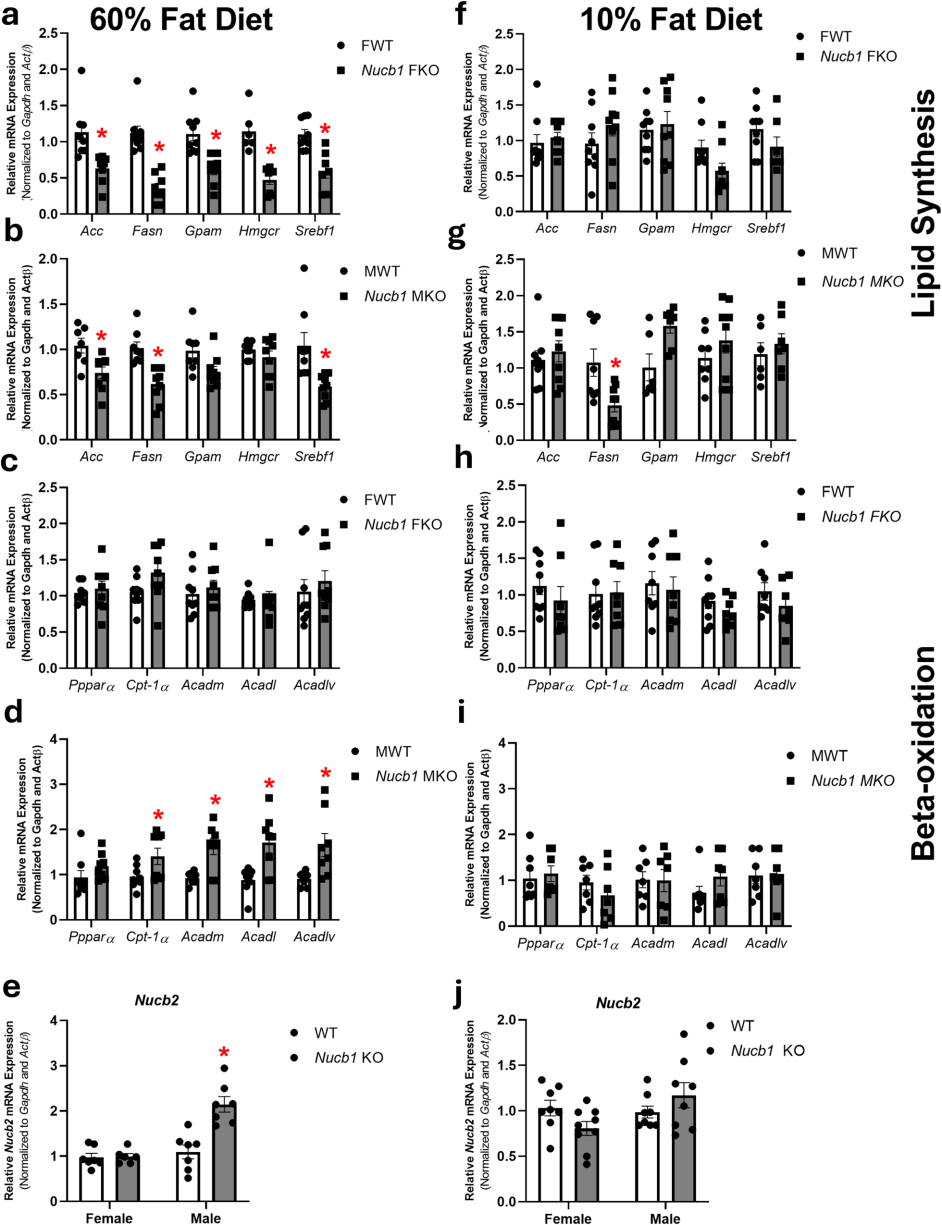
*NUCB1* disruption caused changes in lipid metabolism-regulating enzyme mRNAs. The genetic disruption of *Nucb1* affected lipid pathways-related enzymes (**a**–**d** = 60% fat diet-fed; **f**–**i** = 10% fat diet-fed) and *Nucb* mRNAs (**e** = 60% fat diet-fed; **j** = 10% fat diet-fed) in the liver of mice fed 60% fat diet (left panel), or 10% fat diet (right panel) in a sex- and diet-specific manner. All data are represented as mean ± SEM. The results presented are pooled from at least 7 liver samples (from individual mouse) per group. Asterisks show significant differences between knockout (male/M *Nucb1* or or female/F *Nucb1*; gray bar) and wild-type (WT; white bar) groups. Significance was set at *P* < 0.05. mRNAs measured are: Acetyl-CoA carboxylase (*ACC)*, glycerol-3-phosphate acyltransferase *(GPAM)*, sterol regulatory-element binding factor (*SREBF)-1*, carnitine palmitoyl transferase (*CPT)-1α*, 3-hydroxy-3-methylglutaryl-coA reductase (*HMGCR)*, fatty acid synthase (*FASN)*, diacyl glycerol acyltransferase (*DGAT) 1*, *DGAT2* and long/very long-chain acyl coA dehydrogenase *(ACADL; ACADLV)*, peroxisome proliferator-activated receptor (*Ppar) γ*, acyl CoA dehydrogenase medium chain (ACADM).

The genetic disruption of *Nucb1* downregulated the expression of lipogenic genes (*Acc*, *Fasn*, *Gpam*, *Hmgcr*, *Srebf-1*) in female mice fed 60% high-fat diet (Fig. 7a) and showed a similar pattern in male mice (Fig. 7b), causing a decrease in *Acc*, *Fasn*, and *Srebf-1* mRNA abundance. Although the disruption in *Nucb1* gene did not affect beta-oxidation-related genes in female mice (Fig. 7c), it upregulated the expression of some of these genes (*Cpt-1α*, *Acadl*, *Acadlv*, and *Acadm*) in male mice (Fig. 7d). The difference in the expression of lipogenic and beta-oxidation genes shows a sex-specific effect of *Nucb1* and *Nucb2* gene disruption. Meanwhile, the expression of neither lipogenic genes nor beta-oxidation genes were affected by the disruption of *Nucb1* or *Nucb2* in male or female mice fed 10% fat diet (Fig. 7f–i). The only exception to this was a reduction in *Fasn* in male *Nucb1-*disrupted mice (Fig. 7g). The genetic disruption in *Nucb1* resulted in the upregulation of *Nucb2* mRNA in male mice fed 60% fat diet (Fig. 7e), but not in female mice (Fig. 7e). However, such a change in Nucb2 mRNA was not observed in the same strains of male or female mice fed 10% fat diet (Fig. 7j).

Three-way ANOVA analysis showed that the main effect of “diet” and “gene disruption” on lipogenic enzyme transcripts in *Nucb1-*disrupted mice was significant. In this regard, the two-way interaction of “diet × gene disruption” on these transcripts in both groups was also statistically significant. Furthermore, the significant effect of two-way interactions “diet × sex” and “sex × gene disruption” were observed for some lipogenic genes expression in *Nucb1* gene-disrupted mice. Three-way ANOVA analysis demonstrated that the main effect of “diet” and “gene disruption” on beta-oxidation-related gene expression were significant in the Nucb1-disrupted group. The interaction of “diet × gene disruption” for *Nucb1* group was statistically significant.

### The genetic disruption of *Nucb1* increased the ratio of P-AMPK α/T-AMPK α in mice liver in a sex-specific manner

**Fig. 8:**
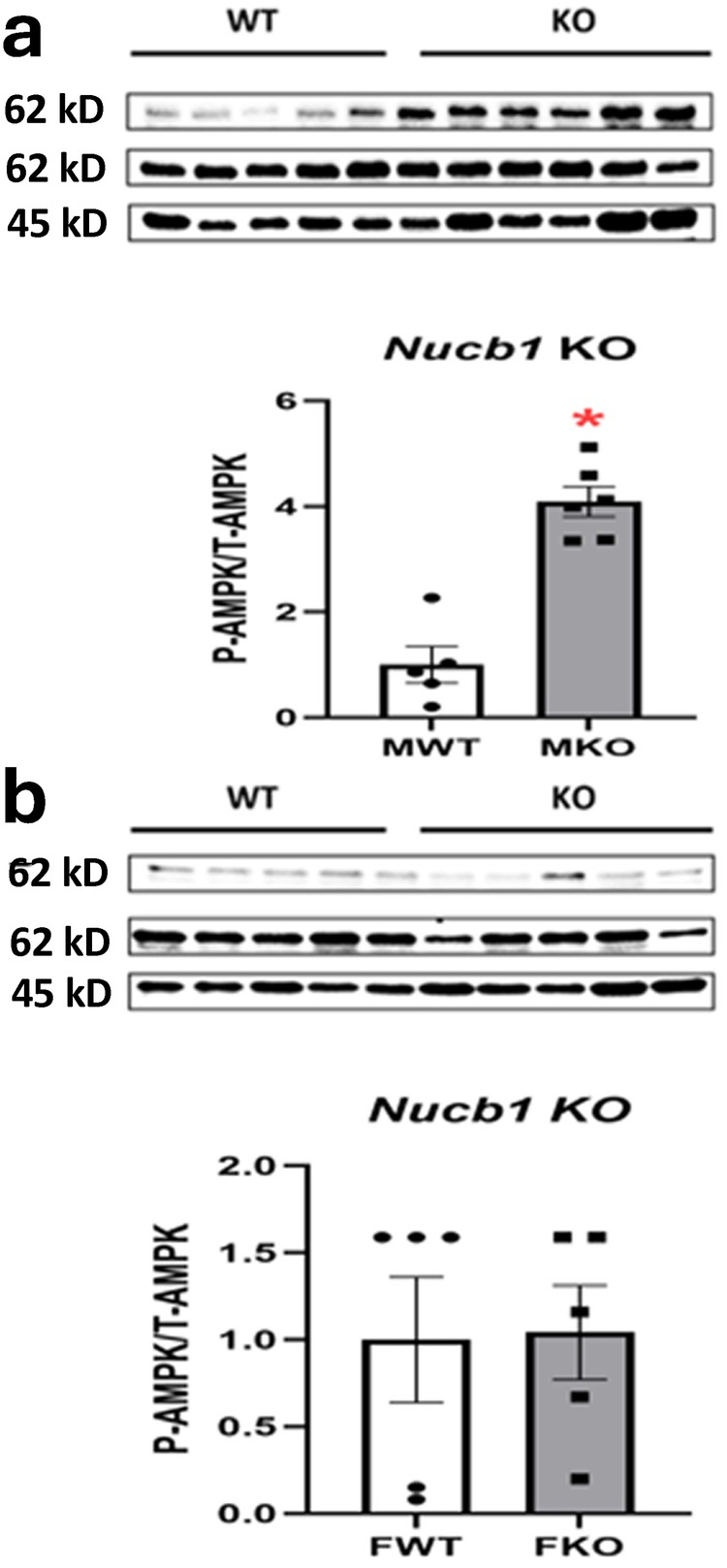
*NUCB1* disruption stimulated P-AMPK α/T-AMPK α ratio. The genetic disruption of nucleobindin 1 *(Nucb1)* increased the ratio of P-AMPK α/T-AMPK α in male mice liver (**a**), but not in female mice liver (**b**). The results presented are pooled from five liver samples per group. Asterisks show significant difference between knockout (KO) and wild-type (WT) groups. Significance was set at *P* < 0.05.

The genetic disruption of *Nucb1* increased the ratio of hepatic P-AMPK α/T-AMPK α in the male mice fed high-fat diet (Fig. 8a), while there were no significant changes in AMPK ratio in female mice (Fig. 8b). This shows the sex-specific effect for the genetic disruption in *Nucb* genes on lipid synthesis and beta-oxidation-mediated pathways.

## Discussion

In this research, first we assessed whether NUCB1/NLP and NUCB2/NESF-1 are present in human hepatocytes, explored if they act directly to regulate lipid accumulation in untreated and OA-induced HepG2 cells, and elucidated some cellular pathways mediating such effects. NUCB1/NLP-like immunoreactivity was reported in the central nervous system and peripheral tissues, including endocrine cells of the anterior pituitary of rats and mice^21,24^, mouse insulinoma MIN6 cell line and mouse pancreatic beta cells^19^ as well as the testis, ovary and pituitary of goldfish^20^. The immunoreactivity of NUCB1/NLP was primarily found in cell cytoplasm in mammalian central and peripheral tissues^21,24,25^. In contrast, NUCB1/NLP was mainly detected in nucleus and in some cells in the cytoplasm, which suggests a cell- and tissue-specific pattern of NUCB1/NLP distribution. Similar to NUCB1/NLP, NUCB2/NESF-1 immunoreactivity was observed in both central and peripheral tissues and cells, including rat anterior pituitary and hypothalamic nuclei^24,26^, mouse corticotrophs^21^, human hypothalamus, rodent duodenum, esophagus, liver, intestine and colon^14^. Different in vitro and in vivo studies reported the positive immunoreactivity of NUCB2/NESF-1 in the cytoplasm of neurons originated from rat and mouse brain^21,24,27^, and in the nuclei of endothelial cells of mouse lung^28^. NUCB2/NESF-1-like immunoreactivity was observed in cytoplasmic vesicles of cells in peripheral tissues, including gastric cells^29^ and rat Leydig cells^30^. *NUCB1* mRNA and nesfatin-1 immunoreactivity was reported in embryonic and adult mouse liver, suggesting a local role for these peptides^31^. *NUCB1* and *NUCB2* mRNA and their proteins were upregulated in NESF-1- and NLP-treated HepG2/C3A cells, respectively. We previously reported that the addition of NLP and NESF-1 suppressed endogenous *Nucb* mRNAs in mouse corticotrophs^21^. It is suggestive of a potential negative feedback loop that causes a reduction in endogenous Nucb production when exogenous NESF-1 or NLP are present. These discrepancies in Nucb expression upon nesfatin-1 or NLP treatment reported here and in our previous research suggest species- and cell-specific pattern for the changes in endogenous NUCB production. Together, these results suggest that human hepatocytes are possibly a cell source of endogenous NUCB and processed peptides. The presence of a signal peptide and processing enzymes suggest secretion of NUCBs and processed peptides from hepatocytes. The hepatic secretory profile of these peptides and whether the endogenous peptides might play a role in hepatic lipid synthesis in hepatocytes warrant further consideration.

Previous studies reported the effect of NESF-1 on glucose and lipid metabolism in rodents and in vitro in goldfish primary hepatocytes, suggesting the presence of a functional receptor for this peptide in the liver^7,32^. We further examined how NLP and NESF-1 regulate the expression of enzymes involved in lipid synthesis and lipid oxidation in human HepG2/C3A cells. A clear downregulation of some lipogenic and beta-oxidation genes in untreated HepG2/C3A cells was observed at 24 h post incubation with 0.1 nM NESF-1 or NLP. In the next step, how these two peptides and quercetin affect lipid accumulation in OA-induced HepG2/C3A cells was determined. Interestingly, both peptides at 0.1 nM and quercetin at 10 µM downregulated lipogenic enzyme expression and upregulated the beta-oxidation gene expression at 24 h post incubation. Continuous peripheral administration of NESF-1 significantly downregulated lipogenic genes and transcription factors in liver samples of male C57BL/6J mice fed 60% fat diet, which agrees with our findings on the regulatory effect of NESF-1 on enzyme expression involved in lipid synthesis^7^. Adipose tissue ppar a, fasn, ppam and slc27a1 were significantly reduced in the white adipose tissue of C57BL/6 J mice continuously infused with 2.5 pmol/mouse/hour nesfatin-1 for 2 weeks. Similarly, the abundance of ppar a, srebp 1, acaca, fasn, gpam, dgat1, and dgat 2 were significantly reduced in the liver of nesfatin-1 infused high-fat diet-fed C57BL/6 mice, but not in mice fed regular chow. In addition, Yin and colleagues^7^ conducted in vitro studies very similar to ours, but using primary hepatocyte derived from C57BL/6J mice. In alignment with the results presented here, primary hepatocyte expression of lipogenic genes srebf-1, srebf2, acaca, fasn, gpam, and dgat1 were suppressed by nesfatin-1. Meanwhile, lipid oxidation genes ppar a, cpt-1 a, acadm, acadl, acadvl and atgl were significantly upregulated by nesfatin-1. While our results reproduced these results in human hepatocytes, it also provided evidence for comparable effects for NLP on effects. NESF-1 was also reported to decrease the expression of lipogenic genes, *Acc* and *Fasn*, in primary brown adipocytes of mice^33^. Moreover, NESF-1 treatment significantly decreased *srebf-1* and enhanced *lpl* and *cpt-1α*, which decreased lipogenesis capacity and increased beta-oxidation in the liver of rainbow trout^34^. Therefore, the comparable biological effects of NESF-1 and NLP are likely due to the high degree of amino acid sequence similarity. Quercetin, the positive control used in our research was widely reported to alleviate lipid accumulation in vitro and in vivo by affecting mRNAs and proteins encoding enzymes within the lipid metabolic pathways. A dose-response was observed for quercetin at a range of 0.1–100 µM, which was associated with *SREBF-1* and *FASN* downregulation in vitro lipid accumulation model using HepG2 cells^35^. In our study, the suppressive effect of quercetin on lipogenic genes was observed within the dose range reported in the study mentioned above.

To estimate lipid accumulation, we then stained lipid droplets using ORO dye. ORO specifically stains the TG and cholesteryl oleate, but no other lipids^36^. Since the hallmark of NAFLD is the TG accumulation in the cytoplasm of hepatocytes^37^, ORO staining can histologically evaluate changes in lipid accumulation. Our results showed that cellular TG was almost undetectable in untreated HepG2/C3A cells. The average stained lipid area decreased remarkably in the NLP and NESF-1 treated group relative to the OA-induced control group. To confirm our ORO staining-based results, cellular TG in HepG2/C3A cells treated with NLP, NESF-1 and quercetin was then measured. Consistently, both NLP and NESF-1, along with the positive control, decreased TG accumulation by approximately twofold in OA-induced HepG2/C3A cells. Similar to our results, peripheral infusion of NESF-1 decreased both ORO-based average area and diameter of lipid droplets in the liver as well as plasma and hepatic TG content in diet-induced obese mice^7^. Furthermore, quercetin was shown to affect lipid accumulation in a dose-dependent manner (1–100 µM) evaluated by an ORO-based colorimetric quantitative assay. The onset of the lipid-lowering effect of quercetin started at 10 µM in the previous study^35^ and this is in agreement with the findings reported here. Plasma nesfatin-1 levels were significantly elevated in a rate model of NAFLD^38^. Meanwhile, serum nesfatin-1 levels were found reduced in patients with NAFLD^17^. While species-specific changes in NAFLD nesfatin-1 profile exists, it is evident that one of the proteins changed in NAFLD is nesfatin-1. Whether NUCB1 and NLP also shows a similar NAFLD profiles is unknown. The role of endogenous NUCBs and processed peptides and its possible role in liver lipid metabolism and NAFLD is also currently unclear.

To assess the potential signaling pathway mediating the lipid attenuation effect of NLP, NESF-1, and quercetin, we first checked the phosphorylation of AMPK at different time points. Although studies on NLP and NESF-1 and its biological roles in different species have progressed well, identifying different cellular cascades mediated by NLP and NESF-1, and their receptor are poorly understood. Labeled-NESF-1 and NLP bind to the surface of rat GH3 cells, suggesting that the effects of these two peptides are mediated by a G-protein coupled receptor (GPCR)^24^. It was reported that NESF-1 stimulates the cellular intake of calcium in the rat hypothalamus neuronal cell culture due to its binding to GPCRs. This effect was blocked when a GPCR disruptor was added to the media^39^. Another study proposed GPCR12 as a potential receptor for NESF-1^40^. Our results suggest the possible presence of a functional NESF-1/NLP receptor in hepatocytes. The activation of this receptor may lead to the phosphorylation AMPK and, subsequently, the alternation in the expression of enzymes involved in lipogenesis. Previous studies confirmed that intervention with AMPK activity by pharmacological approaches is linked with lipid metabolism in the liver^41^. Consistent with pharmacological studies, genetic deletion of AMPKβ1 enhanced lipogenesis while decreasing beta-oxidation in primary culture of hepatocytes. Moreover, AMPKα2 is essential for the hypolipidemic and antisteatotic effects of n-3 long-chain polyunsaturated fatty acids effects in mice^42^. Our results also showed that the inhibition of AMPK by compound C resulted in the accumulation of TG in HepG2/C3A cells. NLP, NESF-1, and quercetin failed to decrease lipid accumulation in the presence of compound C in OA-induced HepG2/C3A cells. Consistently, NESF-1, NLP and quercetin activated AMPK in HepG2/C3A cells. In line with our result, NESF-1 failed to decrease TG accumulation in cultured mouse hepatocytes in the presence of compound C, an AMPK inhibitor^7^. Meanwhile, AICAR, a selective activator of AMPK reduced triglyceride contents in liver cells. Another study revealed that infusion of NESF-1 into the third cerebral ventricle of high-fat diet-fed rats increased hepatic insulin sensitivity through the AMPK-mediated pathway^43^. Moreover, central NESF-1 activated fatty acid oxidation in the muscle through the AMPK-mediated pathway in mice with streptozotocin-induced type 2 diabetes mellitus^44^. Consistent with our results, in vitro and in vivo studies showed that quercetin improves lipid and glucose metabolism through the AMPK-mediated pathway^23,45,46^. Altogether, an unknown GPCR for nesfatin-1/NLP acting through an AMPK-mediated pathway appears to mediate NLP and NESF-1 effects on lipid accumulation in human HepG2/C3A cells.

In the next step, we assessed whether the disruption of *Nucb1* gene affects the expression of enzymes involved in lipid synthesis and oxidation. We also examined the effect of sex, *Nucb1* absence and diet on the expression of genes involved in lipid synthesis. Previous studies reported that feeding a high-fat/high fructose diet resulted in marked sex- and strain-specific changes in the expression of genes involved in lipid synthesis in the liver of male and female mice, with all four hepatic lipid metabolic pathways altered^47^. Another study reported sexual dimorphism of hepatic cholesterol metabolism in exposure to dietary cholesterol in high-fat-fed mice^48^. The results of the current study show that feeding a high-fat diet resulted in changes in gene expression patterns across all mouse groups; however, the severity of these effects was dependent on either sex, *Nucb1* gene disruption or the compensatory upregulation the other *Nucb*. However, lipogenic and beta-oxidation gene pattern in in vitro studies showed a similar pattern where both *NUCB* genes exist in HepG2/C3A cells. These results show that other regulatory signals beyond liver may control lipid metabolism, such as plasma lipid profile, neuroendocrine signals, lipid metabolism state in adipose tissue and muscles. A direct comparison of the in vivo study results to that from the in vitro studies is more complicated due to two reasons. First, the endocrine and metabolic milieu in these mice is multifactorial. Thus, the direct actions of nesfatin-1/NLP shown in human hepatocytes is not comparable to the mouse studies. Species-specific differences also exist, and the example of circulating nesfatin-1 levels in NAFLD in humans and rats discussed earlier is one example for that. In addition, the global changes that occurs due to embryonic disruption of one gene might alter this milieu as well as compensatory changes in other lipid regulators. Therefore, the changes noted in the knockout mice are multifactorial, not solely due to the disrupted gene. Furthermore, the genetic disruption in *Nucb1* upregulated *Nucb2* in a sex-specific manner. This suggests a compensatory response in the upregulation of one *Nucb* during the genetic interruption of the other *Nucb*. Consistently, upregulation of AMPK phosphorylation was observed in *Nucb1* gene disrupted male mice but not in female mice which might be due to the upregulation of endogenous *Nucb* in male mice. Overall, the genetic disruption of *Nucb1* modulates hepatic lipid metabolism in a sex- and diet-specific manner.

**Fig. 9:**
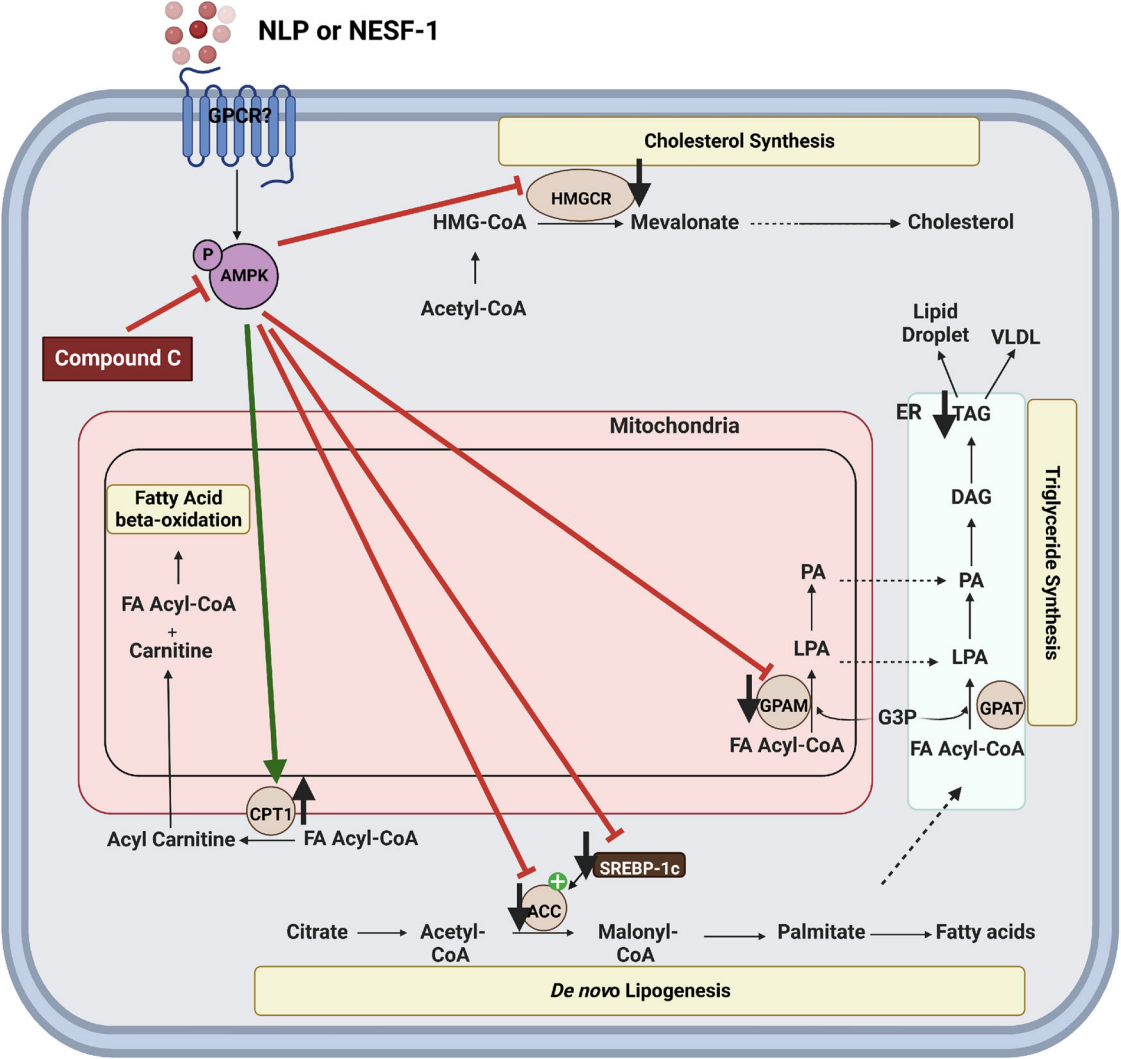
Scheme summarizing nesfatin-1 and NLP effects on hepatocyte lipid metabolism. This figure summarizes the effects of nesfatin-1 and NLP on hepatocyte lipid metabolism, detailed in this manuscript. Nesfatin-1 and NLP bind to a yet-to-be-characterized membrane-bound receptor and elicit their negative (suppressive) effects on cholesterol synthesis, triglyceride synthesis, and de novo lipogenesis. Meanwhile, both nesfatin-1 and NLP increased fatty acid oxidation enzyme mRNAs. Overall, both nesfatin-1 and NLP elicited a lowering of lipid levels. These actions are mediated through the AMPK pathway. Enzymes shown are Acetyl-CoA carboxylase (*ACC)*, glycerol-3-phosphate acyltransferase *(GPAM)*, sterol regulatory-element binding factor (*SREBF)-1*, AMP kinase (AMPK), 3-hydroxy-3-methylglutaryl-coA reductase (*HMGCR)*, and glycerol-3-phosphate acyltransferase (GPAT). This original figure was created using tools provided by Biorender, under a subscription purchased by the corresponding author.

In summary, our results provide the first scientific evidence supporting the direct regulation of hepatic lipid metabolism by NLP and NESF-1 through an AMPK-dependant manner in HepG2/C3A cells (Fig. 9). Genetic disruption of *Nucb1* gene affects lipid metabolism-related gene expression in a sex- and diet-specific manner. This report expands our understanding of NLP and NESF-1 as novel lipid-regulatory peptides in humans and rodents. The receptor that mediates NLP and NESF-1 action, the role of NLP on lipid metabolism in adipose tissue and studying the role of endogenous NUCB in hepatic lipid metabolism remain unknown. The outcome of our study set the stage to pursue a novel strategy targeting NUCB for addressing NAFLD. The results presented here also opens avenues for future research. For example, all studies here are conducted using a human liver cell line. The repetition of these experiments in human liver primary cells is one important next step. Additional studies to test whether nesfatin-1 and NLP affect metabolic fluxes and de novo lipogenesis as well as beta-oxidation fluxes warrant attention. In addition, in vivo studies to determine if the lipid-lowering effects, especially for NLP found in vitro exist in vivo in animal models of NAFLD.

## Methods

### Cell culture

Human hepatocellular carcinoma cells (HepG2/C3A cells, Catalog # CRL-1074, ATCC, USA) were grown as per protocol provided by the supplier, in 10 cm cell culture dish containing in 10 mL DMEM supplemented with 10% FBS at 37 °C in 5% CO_2_ in 95% humidified atmosphere. HepG2/C3A cells are derived from HepG2 (ATCC HB-8065 is an epithelial-like cell isolated from the liver of a 15-year-old male patient with hepatocellular carcinoma. When cells reached the required confluency, they were used for specific experiments.

### Immunocytochemistry

After seeding 1 × 10^8^ cells/ 0.5 mL/well of foue-well of chamber slides and cells reaching at 40% confluency, they were fixed by 4% paraformaldehyde (PFA) (Catalog # 158127, Sigma Canada) and washed with 1× PBS. Next, cells were incubated with a series of PBS-based solutions, including PBS-PF (Catalog # 1464510, Kodak, Canada), PBS-Triton X-100 (Catalog # 9036-19-5; Millipore Sigma Canada) and PBS-ABB (Pierce Catalog # 54200, Thermo Scientific, Canada), respectively. After incubation of cells with primary antibody overnight at 4 °C, they were incubated with secondary antibody at room temperature for 1 h and subsequently were washed with PBS-based solutions. Finally, chamber walls were removed, followed by the addition of Vectashield DAPI containing mounting medium (Catalog # H-1200-10, Vector Labs, California, USA) to cells, and then glass slides were applied to mount slides. All images were taken by Olympus DP70 camera and Olympus BX51 microscope (Olympus Canada, Toronto, Canada).

The primary antibodies used in this study were rabbit anti-mouse NUCB1 (1:200, custom synthesized, cat no. 1312-PAC-02, Pacific Immunology, USA), rabbit anti-mouse NUCB2 (1:200, RRID: AB_2891124, cat no. 1312-PAC-01, Pacific Immunology, USA). Secondary antibodies in this study were goat anti-rabbit Alexa Fluor 488 (1:200, RRID: AB_2630356, cat no. ab150077, ABCAM, UK). No primary antibody treated group was included as a negative control. Immunostaining obtained is referred to as NUCB1/NLP and NUCB2/NESF-1 to represent precursors and the encoded peptides detected by the primary antibodies used.

### Induction of lipid accumulation in HepG2/C3A cells using OA

HepG2/C3A cells at approximately 60% confluency cultured in FBS-free medium (Thermo Scientific Canada) in four-well chamber slides were exposed to nontoxic concentration range of BSA conjugated-OA (O3008, Sigma-Aldrich®, Darmstadt, Germany) for 48 h and then lipid intensity area was measured by ORO staining. The concentration of OA and time point was chosen based on previous publications^49,50^. The effective dose of OA was chosen based on ORO-based results.

### Testing the effects of NLP and NESF-1 on lipid pathways-related enzymes mRNAs and endogenous *NUCB* and NUCB in untreated and OA-induced HepG2/C3A

HepG2/C3A cells at a confluency of 90% were incubated with 0.1 nM of mouse NLP (custom synthesized peptide, >95% purity, ABGENT, USA) or rat NESF-1 (cat no. 003-22B, Phoenix Pharmaceuticals Inc. CA, USA) for 24 h. The effective dose and time point were decided based on our pilot studies. To induce lipid accumulation, HepG2/C3A cells at a confluency of 50–60% were incubated with 0.5 mM BSA conjugated-OA for 48 h, then media refreshed with either rat NESF-1 or mouse NLP for another 24 h. Finally, RNA samples were extracted to assess the target transcript abundance by real-time quantitative PCR (qPCR). For OA-induced HepG2/C3A cell studies, quercetin (cat no. Q4951, Sigma-Aldrich, Darmstadt, Germany) was chosen as a positive control group. Quercetin doses were selected based on previous scientific findings^51^. Quercetin was dissolved in DMSO (Catalog # 136321-15-8; Millipore Sigma, Canada) as stock solution and then diluted in cell culture media (final concentration below 0.1%). The abundance of endogenous *NUCB* and NUCB levels was also assessed in OA-induced HepG2/C3A cells.

### ORO staining

A stock solution of 0.5% ORO (Allied Chemical, Morristown, NJ, USA) was prepared, and it was further diluted with sterile water (ratio 3:2) and filtered to make a working solution. OA-induced HepG2/C3A cells seeded in 4-well chamber slides at a confluency of 90% were incubated with an effective dose of rat NESF-1 or mouse NLP or quercetin for 24 h. No OA-treated controls (untreated groups) were also included in these studies. After fixing the cells in 4% paraformaldehyde (PFA), they were washed twice with 1× PBS. Chamber slide walls were removed, and slides were stained with ORO working solution for 15 min at room temperature. After rinsing with 60% isopropanol and subsequently in distilled water, they were mounted in glycerine jelly. Finally, sections were visualized using an Olympus BX51 microscope connected to an Olympus DP70 camera in light mode.

### Mechanism of action of NLP and NESF-1 on HepG2 cells

A specific AMPK inhibitor, compound C (cat no. ab120843, ABCAM, UK) was used to block AMPK-mediated pathway in HepG2/C3A cells. Briefly, OA-induced cells at a confluency 90% were preincubated with compound C (10 µM) for 1 h, followed by incubation with peptides (0.1 nM) or quercetin (10 µM), and compound C. Compound C stock was dissolved in DMSO and the level of DMSO in cell culture media was less than 0.1%. The concentration and time point for compound C incubation was chosen based on the recommended ranges found in the supplier catalog and were independently validated in pilot studies. Following the incubation, samples were collected for intracellular measurements as described below.

### Measurement of cellular triglycerides

OA-induced HepG2/C3A cells were incubated with media containing the specified dose of peptides, positive control, and inhibitors in the six-well plate for 24 h, and then cells were used for lipid extraction using the hexane-isopropanol method with modifications^52,53^. The groups consisted of media alone, NLP, NESF-1, quercetin, compound C, NLP + compound C, and NESF-1+compound C. Briefly, cells were washed with cold 1× PBS twice and then were scarped from the bottom of a six-well plate, followed by transferring into the new tubes. After centrifugation at 13,000 × *g* for 10 min, the supernatant was removed, and cell pellets were resuspended in hexane:isopropanol (3:2, v-v). This was followed by centrifugation using the same setup, and the supernatant was transferred to new tubes and dried at 50 °C overnight. Finally, dried viscous pellets were dissolved in isopropanol: NP-40 alternative (cat no. 492018, Sigma-Aldrich®, Darmstadt, Germany) (9:1, v- v) at 50 °C for 5 min. These samples were used to measure cellular TG content using Infinity TG liquid stable reagent (cat no. TR22421, Thermo Fisher Scientific, USA). For TG measurement, 3 µL of extracted samples were mixed with 300 µL of reagent per well of a 96-well plate at 37 °C for 5 min, and then the absorbance of each well was measured in primary and secondary wavelengths of 500 and 660 nm using a Varioskan LUX multimode microplate reader (Thermo Fisher Scientific, USA). TGs were calculated based on the formula mentioned in the instruction of the reagent as mentioned below:

TGs (mmol/L) = (absorbance of unknown)/(absorbance of calibrator) × calibrator value.

### Animals

To characterize the effects of the genetic disruption of *Nucb1* in the lipid metabolism, breeding pairs of homozygous C57BL/6NCrl-^Nucb1em1(IMPC)Mbp/Mmucd^ mice from the University of California, San Diego (generated by Dr. Kent Lloyd, Mouse Biology Program, University of California-Davis, USA) were used to establish a colony of Nucb1-disrupted mice. To generate these global Nucb1-disrupted mice, the exon 4 and flanking splicing regions of *Nucb1* were constitutively deleted using CRISPR Cas9 gene editing technology in C57BL/6 J mouse zygotes. Further details on the phenotype of this strain of mice are furnished in ref. ^54^.

All animals were housed under 12 h light:12 h dark cycle (7 am–7 pm), humidity (30–60%), and temperature (18–22 °C) controlled vivarium located in the Western College of Veterinary Medicine Animal Care Unit, University of Saskatchewan. All *Nucb1* KO mice studies followed the guidelines of the Canadian Council for Animal Care and were approved by the University of Saskatchewan Animal Care Committee (2012-0033). Age-matched mice were chosen and fed a control fat diet (Cat no. D12450B, Research Diets Inc., 70% carbohydrate, 20% protein, 10% fat, Energy density = 3.82 Kcal/g) and high-fat diet (cat no. D12492, Research Diets Inc., 20% carbohydrate, 20% protein, 60% fat, Energy density = 5.21 Kcal/g) for 6 months. Liver samples from these mice were collected after cervical dislocation, total RNA was extracted, and the expression of liver genes was assessed.

### RNA extraction, cDNA synthesis, and RT-qPCR

The above procedures were carried out as described in our previously reported protocols^21^. Briefly, RNA was extracted using Ribozol (cat no. N580, VWR, USA), and then RNA’s quantity and quality were determined by NanoDrop 2000 (Thermo Fisher Scientific). The RNA was reverse transcribed to the cDNA using iScript Reverse Transcription Supermix for RT-qPCR (cat no. 170884, Bio-Rad, USA) followed by the quantitative measurement of mRNA expression by qPCR in a CFX Connect Optic module (Bio-Rad, USA) following the requirements of the MIQE guidelines^55^ and using SensiFAST™ SYBR No-ROX MIX (cat no. BIO-98050, Bioline, UK). The primers sequence and annealing temperature are listed in Table S1 (Supplementary Table 1) purchased from Integrated DNA Technologies (IDT). Three negative controls, including no template DNA (NTC control), no reverse transcriptase control from cDNA synthesis process (RTC control), and a nuclease-free water sample (PCR control), were also included for each gene expression study. Thermal cycling setup for all genes was the following: denaturation (95 °C for 5 s), annealing (specific for each primer for 25 s) and elongation (60 °C for 25 s), 35 cycles. At least three independent experiments with triplicates for in vitro studies (final samples ≥9) and more than five mice/group/sex were considered. The abundance of mRNA was calculated based on the Pfaffl method and gene-specific efficiencies^56^, relative to the geometric means of the 2 housekeeping genes. Four different housekeeping genes (Beta actin, GAPDH, HMBS, and HMBeta2) were tested in HepG2 cell studies. HMBS and HMBeta2 for HepG2 cell studies had constant Ct values and better stability values, and they were chosen as internal controls.

### Western blot analysis

The analysis of protein expression was done according to the previously reported protocols^21^. Briefly, total protein was extracted using RIPA Lysis, and Extraction Buffer (cat no. 89901, Thermo Fisher Scientific, USA) and the concentration of protein was determined by Bradford assay. Then, 35 μg of crude protein was electrophoresed on 8–16% Mini-Protean TGX gels (cat no. 456-1104, Bio-Rad, USA), and the protein bands were transferred to nitrocellulose membranes (cat no. 1704158, Bio-Rad, USA) using the Trans-Blot Turbo Transfer System (Bio-Rad, USA). After blocking the membrane with rapid blocking buffer 1× (cat no. M325, VWR, USA) for 15 min at room temperature, it was incubated with primary antibodies overnight at 4 °C followed by washing with TBST (0.1% Tween-20 in TBS) thrice for 15 min each time. In the next step, nitrocellulose membranes were incubated with a secondary antibody for 1 h at 37 °C, and final washing steps were completed. The membranes were visualized using a ChemiDoc MP Imaging System (Bio-Rad, USA). The band intensity was analyzed by ImageJ (National Institutes of Health, Bethesda, MD, USA). All blot images are provided as Supplementary Figs. 2–5*.*

Primary antibodies used in this study were monoclonal mouse anti-beta-actin antibody (1:1000, RRID: AB_528068, cat no. JLA20, Developmental Studies Hybridoma Bank, DSHB, University of Iowa, USA), rabbit anti-mouse NUCB1 (1:250, custom synthesized, cat no. 1312-PAC-02, Pacific Immunology, USA), rabbit anti-mouse NUCB2 (1:250, RRID: AB_2891124, cat no. 1312-PAC-01, Pacific Immunology, USA), polyclonal rabbit anti-phospho (Thr172)-AMPKα antibody (1:1000, RRID: AB_330330, cat no. 2531S, Cell Signalling, USA), polyclonal rabbit anti-AMPKα antibody (1:1000, RRID: AB_330331, cat no. 2532S, Cell Signalling, USA). The secondary antibodies used were goat anti-rabbit IgG (H + L)-HRP conjugate antibody (1:5000, RRID: AB_11125142, cat no. 170-6515, Bio-Rad, USA) and goat anti-mouse IgG (H + L)-HRP conjugate antibody (1: 5000, RRID: AB_11125547, cat no. 170-6516, Bio-Rad, USA).

### Statistical analysis

All values are shown as mean ± SEM. Statistical analysis was conducted by SPSS statistical software (IBM SPSS Statistics for Windows, Version 23.0, USA). The normality of data and the presence of outliers were assessed by Shapiro–Wilk’s test and drawing box plot, respectively. The homogeneity of variances was analyzed by Leven’s test. The single comparison was performed by Student’s *t* test. Multiple comparisons were done by one-way ANOVA followed by Tukey’s multiple comparison test. The three-way ANOVA was performed to evaluate the main effect or combination of three independent variables (diet, sex, gene disruption) on response variables (lipogenic or beta-oxidation gene expression) in mice. The significance level was set at *P* < 0.05. Graphs were plotted by GraphPad Prism (GraphPad Software, Inc., Prism 8 for Windows, Version 8.4.2, USA).

### Reporting summary

Further information on research design is available in the Nature Portfolio Reporting Summary linked to this article.

### Supplementary information

Supplementary Material

Description of Additional Supplementary Files

Supplementary Data 1

Reporting summary

## Data Availability

Extra information supporting the findings of this study and primer sequences are available within its supplementary information file. The raw data that support the findings of this study are provided as Supplementary Data 1 in MS Excel format.
