# Supplementary material for: Nesfatin-1 and nesfatin-1-like peptide attenuate hepatocyte lipid accumulation and nucleobindin-1 disruption modulates lipid metabolic pathways
Source: Commun Biol. 2024 May 27;7:623. doi: 10.1038/s42003-024-06314-2 (PMC11130297; doi:10.1038/s42003-024-06314-2)
Supplement: Supplementary file 1 — Supplementary Material [file 42003_2024_6314_MOESM1_ESM.docx]

**Nesfatin-1 and nesfatin-1-like peptide attenuate hepatocyte lipid accumulation and nucleobindin-1 disruption modulates lipid metabolic pathways**

Atefeh Nasri ^1^, Mateh Kowaluk^1^, Scott B Widenmaier^2^, Suraj Unniappan^1^

^1^Laboratory of Integrative Neuroendocrinology, Department of Veterinary Biomedical Sciences, Western College of Veterinary Medicine, University of Saskatchewan, Saskatoon, Saskatchewan S7N 5B4, Canada

^2^Department of Anatomy, Physiology and Pharmacology, College of Medicine, University of Saskatchewan, Saskatoon, Saskatchewan S7N 5E5, Canada

**Supplementary Material**

**Table S1. Primer sequences**

**Figure S1. No-primary antibody-negative controls**

**Figure S2. Blot for AMPK and beta actin in HepG2 cells**

**Figures S3 and S4. Blots for NUCB 1 and NUCB2 in HepG2 cells**

**Figure S5. Blots for AMPK in mice liver samples**

| **Table S1**. The list of primer forward and reverse sequences and their annealing temperature | | | | |
| --- | --- | --- | --- | --- |
| Gene | Accession no. | Primer sequence (5′−3′) | | Temp (°C) |
|  |  | Forward | Reverse |  |
| HM-*FASN* | XM_011523538.3 | GACACAGTCACCATCTCGGG | TTGGCAAACACACCCTCCTT | 60 |
| HM-*ACC* | U19822.1 | CTGTAGAAACCCGGACAGTAGAAC | GGTCAGCATACATCTCCATGTG | 60 |
| HM-*GPAM* | XM_024448089.2 | CGAGTGGATTTTGCACAGCC | CGCTTGCTCCAGGGAAAGTA | 60 |
| HM-*DGAT1* | XM_047422389.1 | GCTTCAGCAACTACCGTGGCAT | CCTTCAGGAACAGAGAAACCACC | 60 |
| HM-*DGAT2* | XM_047427716.1 | AGTGGGTCCTGTCCTTCCTT | TCTTGGGTGTGTTCCAGTCA | 60 |
| HM-*HMGCR* | XM_011543358.2 | TTCGGTGGCCTCTAGTGAGA | GATGGGAGGCCACAAAGAGG | 60 |
| HM-*SREBF1* | BC063281.1 | TGACCGACATCGAAGGTGAAG | TGGGTCAAATAGGCCAGGGA | 60 |
| HM-*PPARΑ* | XM_047441426.1 | GCTGGTGTATGACAAGTGCGA | ATTCGTCCAAAACGAATCGCGT | 60 |
| HM-*CPT1Α* | XM_017017220.2 | CAGGCCGAAAACCCATGTTG | AGGCCTCACCGACTGTAGAT | 60 |
| HM-*ACADM* | NM_001286043.2 | CTGCAGGGTCCTGAGAAGTA | GGTTCACGTTGTCGATTGGC | 59 |
| HM-*ACADL* | NM_001608.4 | TCAGCTGATCGTCCTCCCT | ATGAGAACATCGCGCGGC | 60 |
| HM-*ACADLV* | XM_047435932.1 | CTGTGGCCGCTTTCTGTCTA | TCTGCTAGGCCCCCATTACT | 60 |
| HM-*NUCB1* | XM_017026845.2 | GACCCTCAGAACCAGCATACA | CTCCCAGTGACTCCAGATAACG | 60 |
| HM-*NUCB2* | XM_047426994.1 | AGTAGATGAGGTGGAGGACCA | TAGGCACAGCTTCAAGAGCA | 59 |
| HM-*Β2M* | XM_005254549.4 | CTCCGTGGCCTTAGCTGTG | TTTGGAGTACGCTGGATAGCCT | 62 |
| HM-*MBS* | NM_001258208.2 | TGCAACGGCGGAAGAAAA | ACGAGGCTTTCAATGTTGCC | 60 |
| MS-*Fasn* | XM_030245556.1 | GGCCCCTCTGTTAATTGGCT | GGATCTCAGGGTTGGGGTTG | 60 |
| MS-*Acc* | NM_133360.3 | ATGGGCGGAATGGTCTCTTTC | TGGGGACCTTGTCTTCATCAT | 59.4 |
| MS-*Gpam* | NM_001356285.2 | TTATCACCAGGACGGAAAGG | TTTTCACAGCGTTCTTCACG | 57 |
| MS-*Hmgcr* | XM_036157854.1 | TCAAGGAACGTGCACCAAGA | TCCTTGAGAACCCAATGCCC | 57 |
| MS-*Srebf1* | BC006051.1 | ATGCCATGGGCAAGTACACA | ATAGCATCTCCTGCGCACTC | 59.50 |
| MS-*Pparα* | XM_030248424.2 | TGCAAACTTGGACTTGAACG | GATCAGCATCCCGTCTTTGC | 57.7 |
| MS-*Cpt1α* | XM_036161417.1 | GGGCCATCTGTGGGAGTATG | ACTGTAGCCTGGTGGGTTTG | 59.6 |
| MS-*Acadm* | NM_007382.5 | GAACCAGACCTACAGTCGCAG | AGGGCATACTTCGTGGCTTC | 61.3 |
| MS-*Acadl* | NM_007381.4 | CATTGGTGGGGACTTGCTCT | TGGCTATGGCACCGATACAC | 59 |
| MS-*Acadlv* | NM_017366.3 | TTGGCAGAGATTGTGGGCAT | CTGGGCCTTTGTGCCATAGA | 57 |
| MS-*Nucb1* | XM_006540696.4 | CACGGGCCTGTACTACCAC | TTGGCTCAGCTTTCCACTCT | 60 |
| MS-*Nucb2* | XR_004934144.1 | AACACGAGCGGAGAGAGTAT | AGGGTCCAATCCATCAGTCT | 60 |
| MS*-Actβ* | BC138614 | CCACTGCCGCATCCTCCTCC | CTCGTTGCCAATAGTGATGAC | 60 |
| MS*-Gapdh* | XM_036165840 | GACATCAAGAAGGTGGTG | ATACCAGGAAATGAGCTTGACAAA | 59 |

**
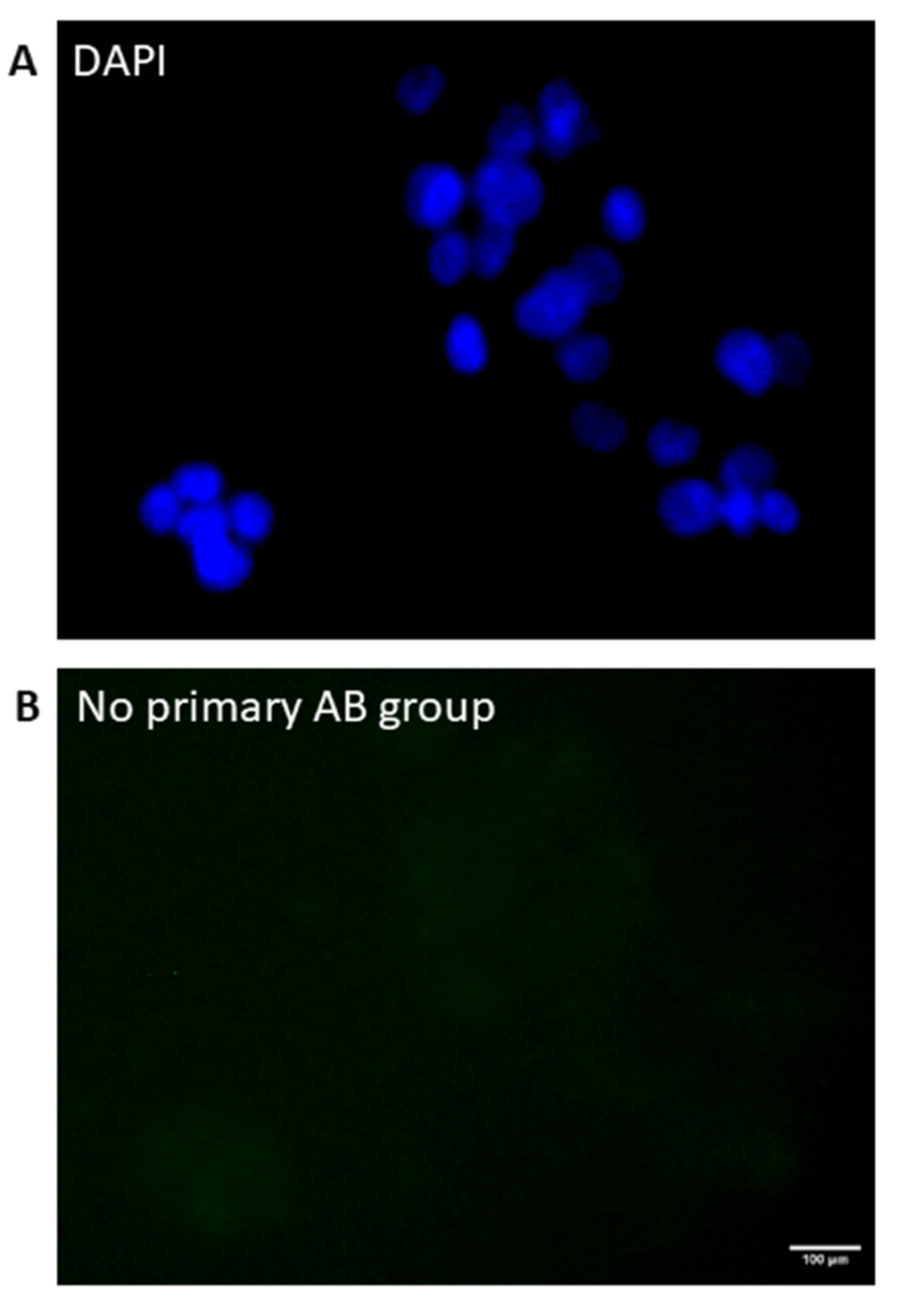
**

**Supplementary Figure 1.** HepG2/C3A cells do not show any immunoreactivity signals in no primary AB treated groups (B). DAPI stained DNA in blue color (A). Secondary antibody: goat anti-rabbit Alexa Fluor® 488 (1:500, RRID: AB_2630356, cat no. ab150077, ABCAM, UK).


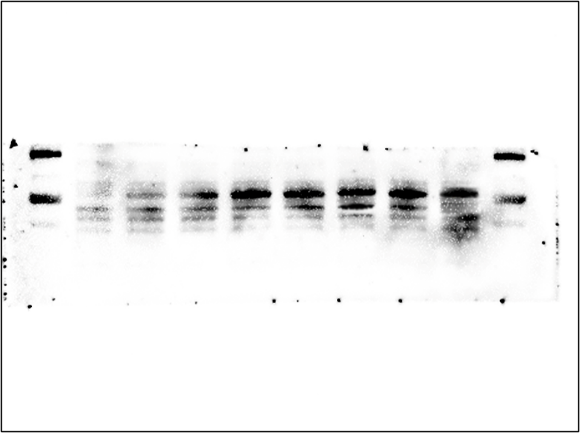

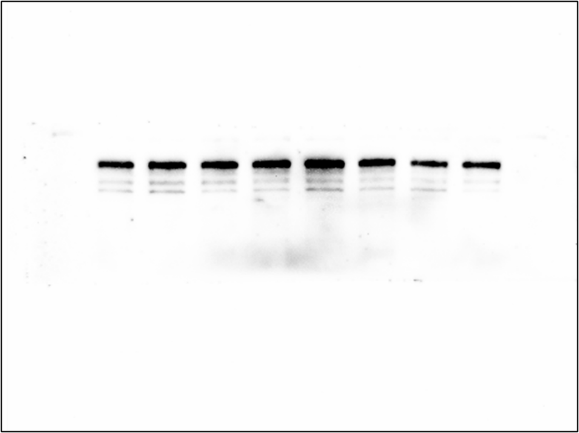


**T-AMPK**

**62kD**

**P-AMPK**

**62kD**


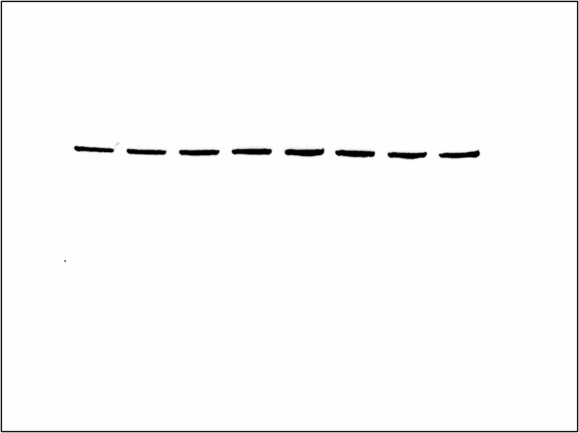
**
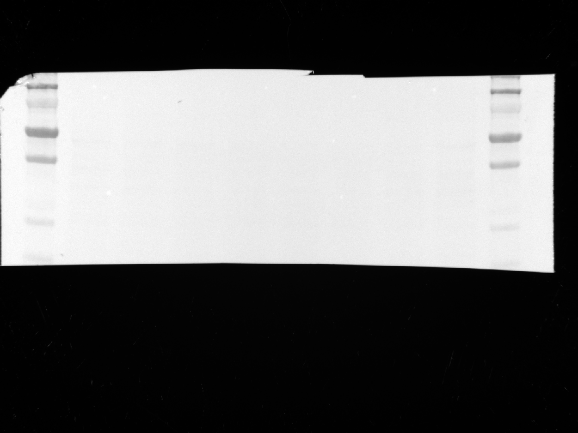
**

**Beta Actin**

**45kD**

50KD

**Supplementary Figure 2.** Blots for P-AMPK, T-AMPK and beta actin in oleic acid-induced HepG2/C3A

**
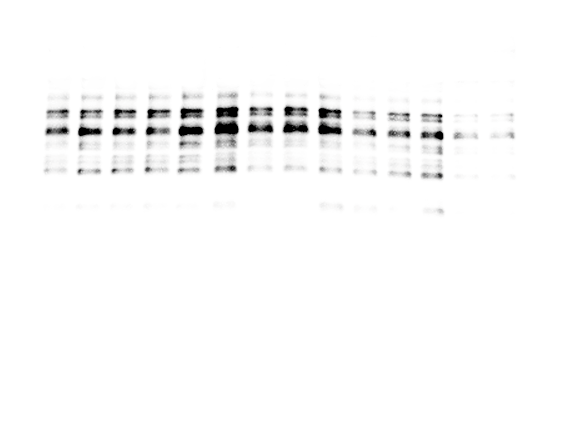

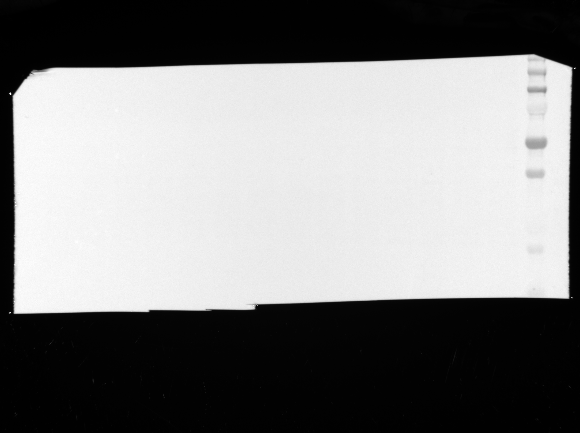

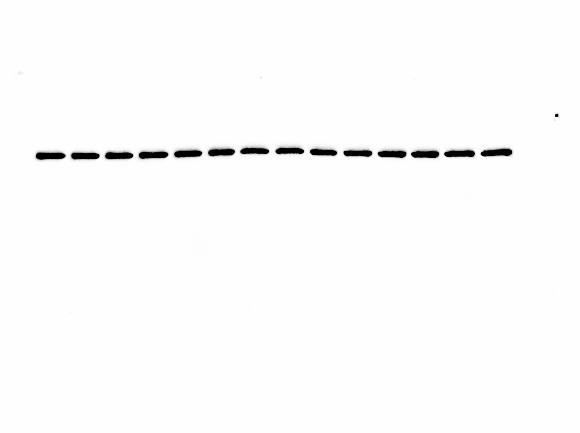
**

50KD

**Beta-ACTIN**

**45KD**

**NUCB1**

**53.5kD**

**Supplementary Figure 3. Blots for NUCB1 and beta actin in HepG2/C3A**

**
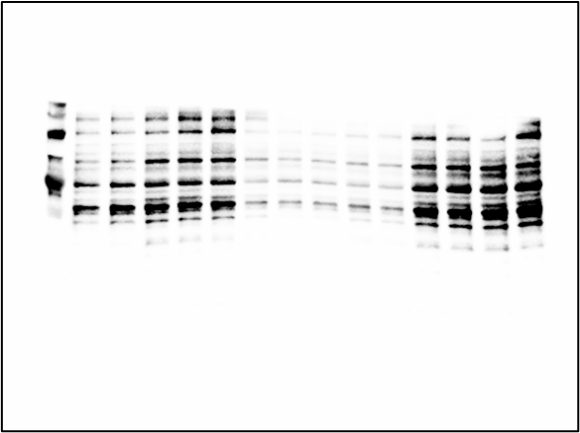

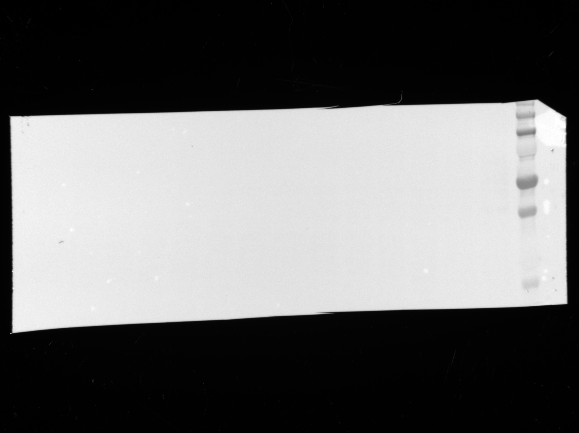

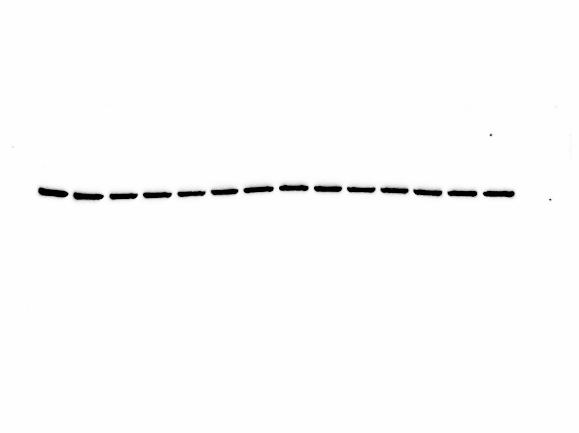
**

50KD

**Beta-ACTIN**

**45KD**

**NUCB2**

**50.1KD**

**Supplementary Figure 4. Blots for NUCB2 and beta actin in HepG2/C3A**

**
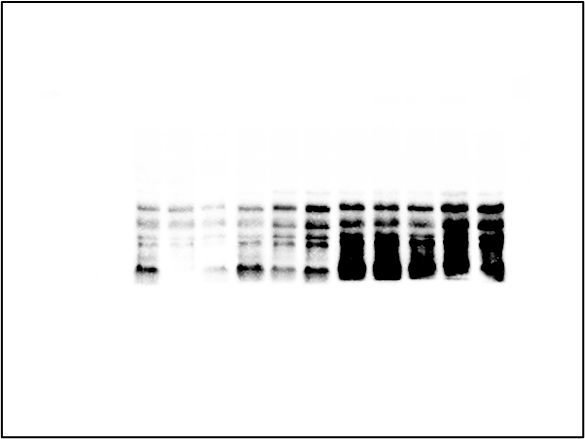

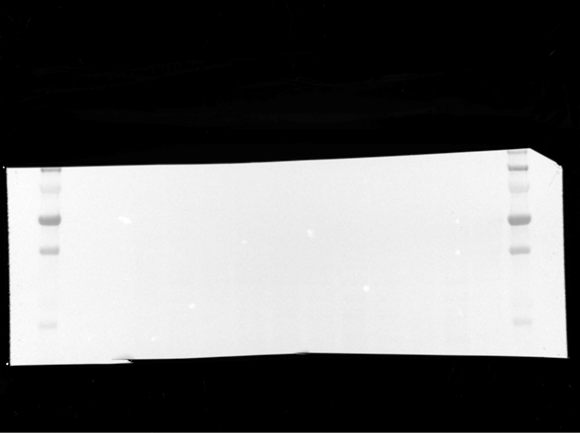
**

50KD

**P-AMPK in male *Nucb1* KO**

**62KD**

**
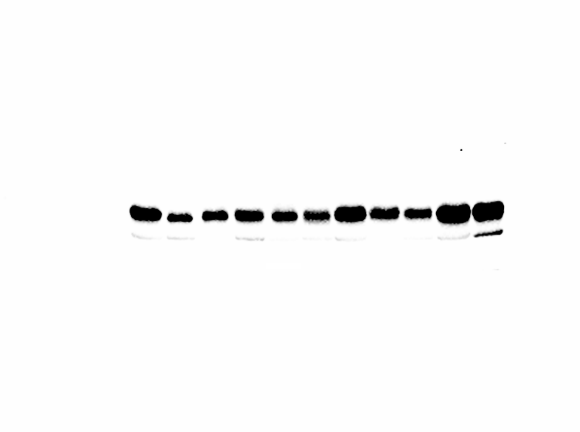

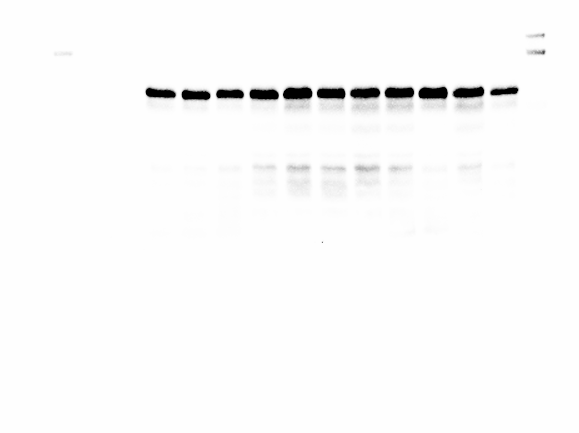
**

**T-AMPK in male *Nucb1* KO**

**62KD**

**Beta-actin in male *Nucb1* KO**

**45KD**


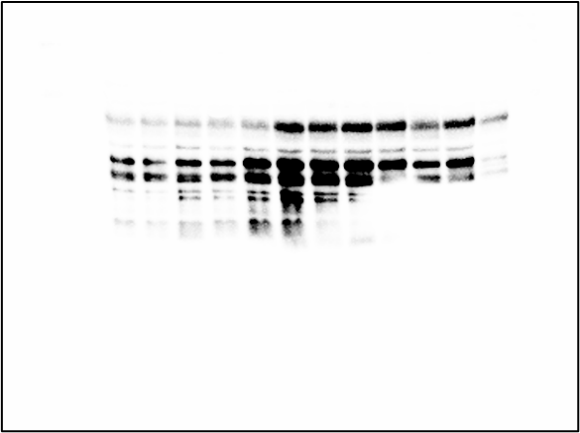

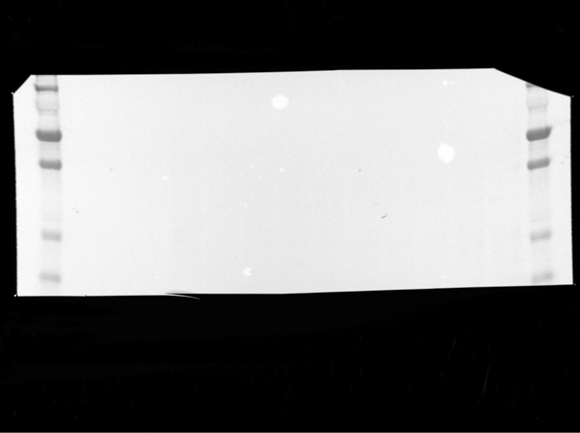


50KD

**P-AMPK in male *Nucb2* KO**

**62KD**

**
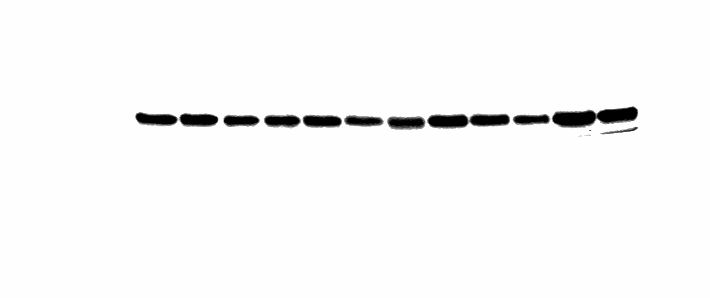
**
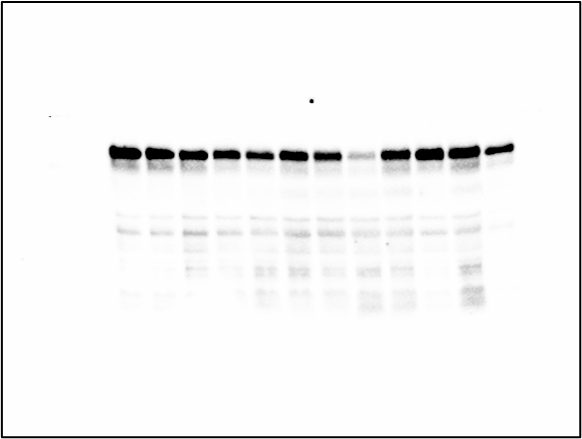


**T-AMPK in male *Nucb2* KO**

**62KD**

**Beta-actin in male *Nucb1* KO**

**45KD**

**
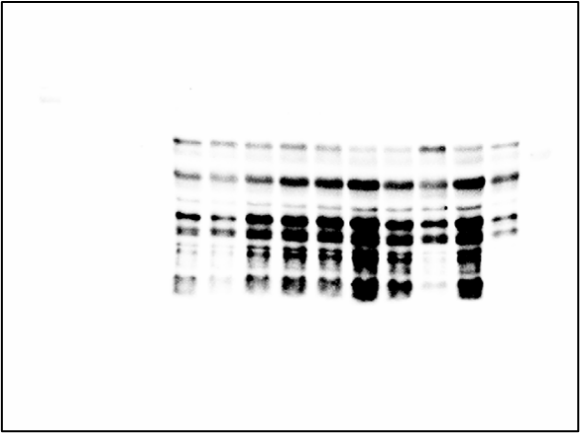

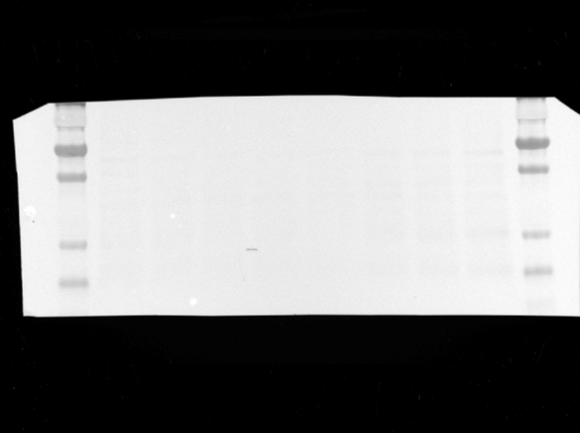
**

50KD

**P-AMPK in female *Nucb1* KO**

**62KD**

**
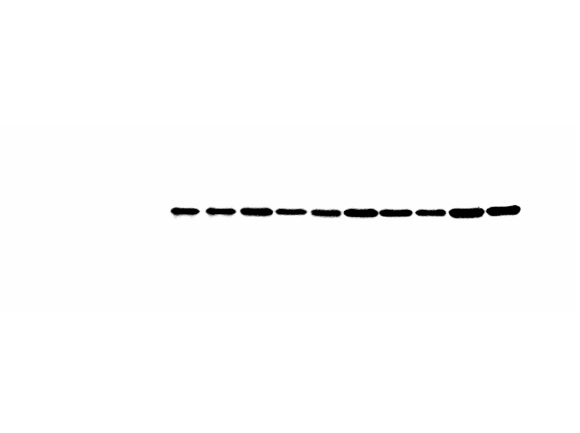

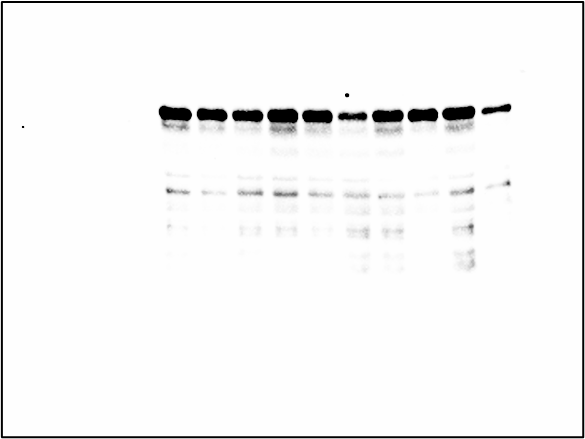
**

**T-AMPK in female *Nucb1* KO**

**62KD**

**Beta-actin in female *Nucb1* KO**

**45KD**

**
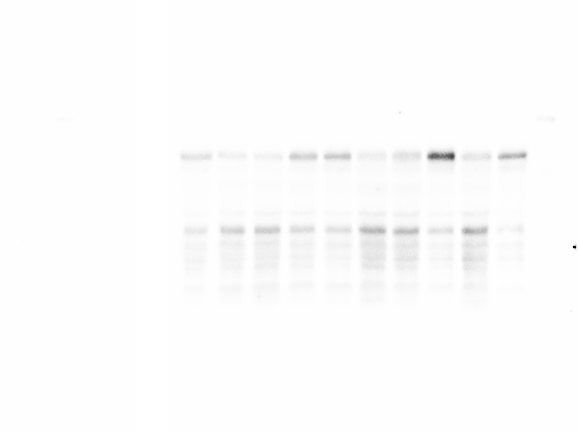

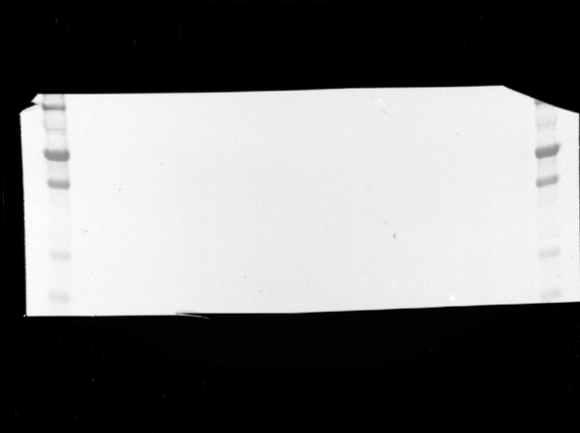
**

50KD

**P-AMPK in female *Nucb2* KO**

**62KD
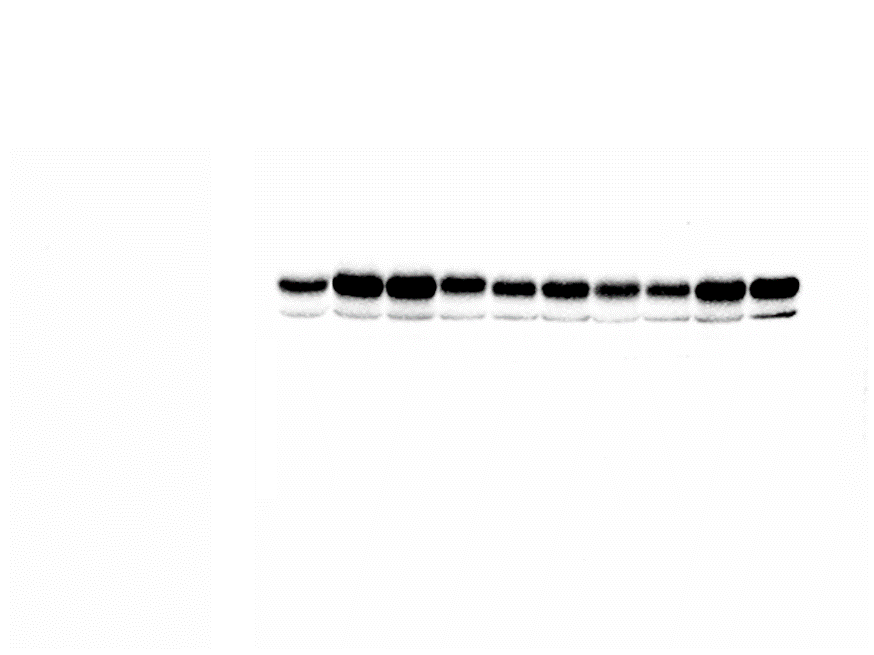
**

**
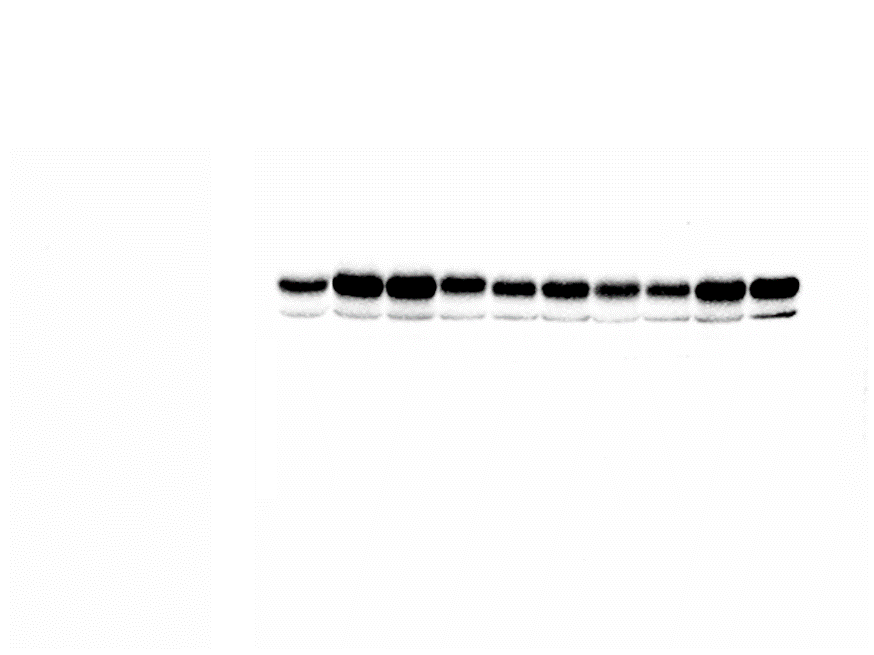

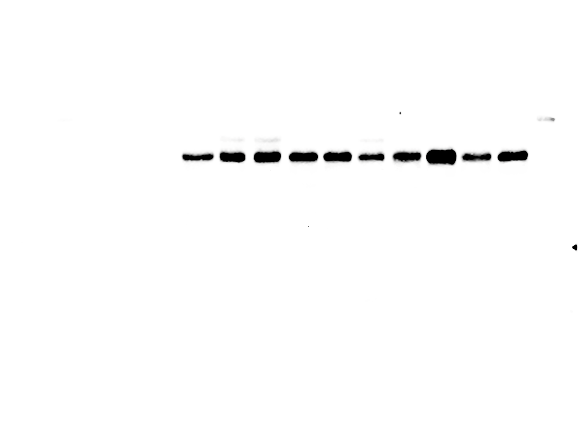
**

**T-AMPK in female *Nucb2* KO**

**62KD
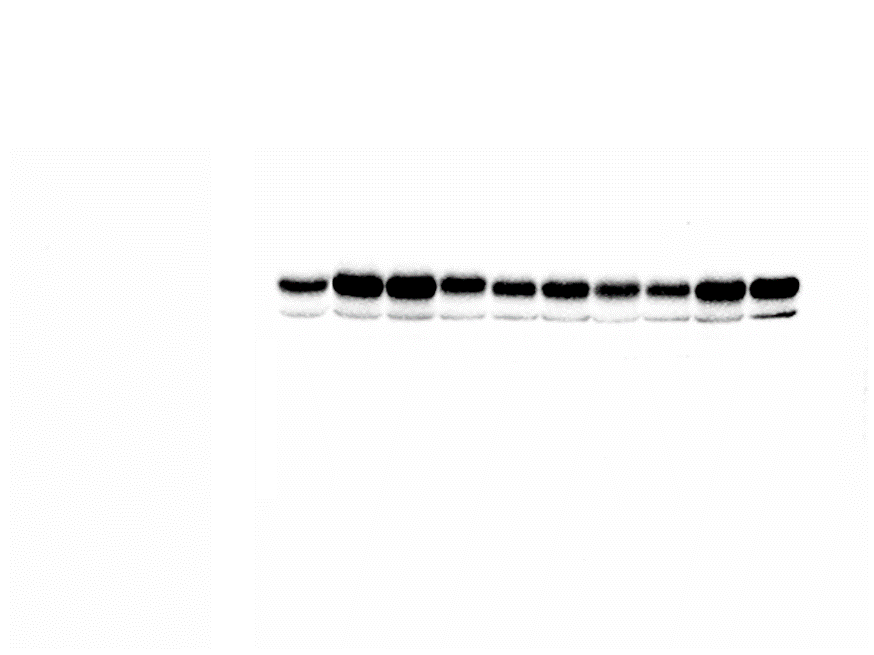
**

**Beta-actin in female *Nucb2* KO**

**45KD
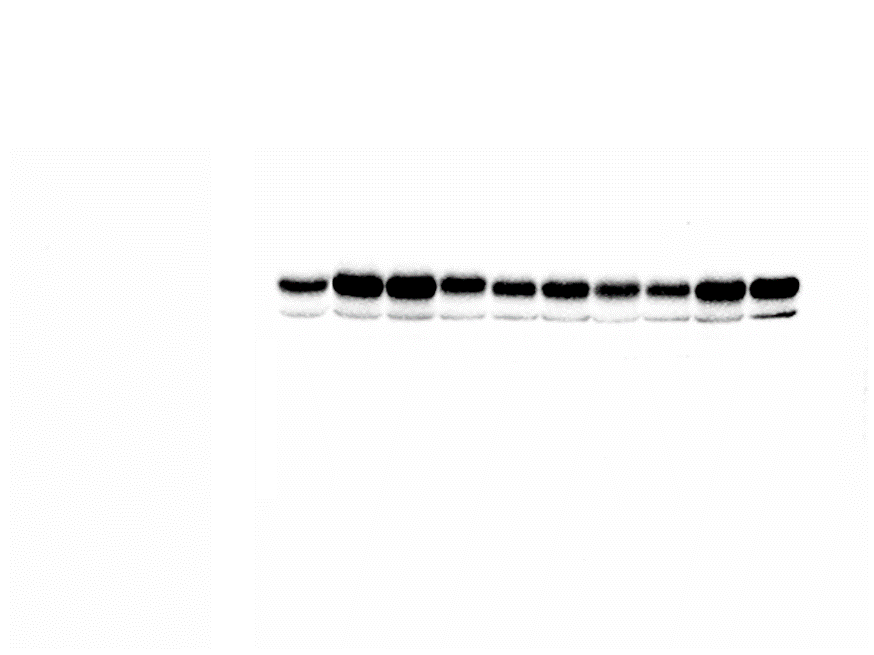
**

**Supplementary Figure 5.** Blots for P-AMPK, T-AMPK and beta actin in mice liver samples
