# Supplementary material for: Nesfatin-1 and nesfatin-1-like peptide attenuate hepatocyte lipid accumulation and nucleobindin-1 disruption modulates lipid metabolic pathways
Source: Commun Biol. 2024 May 27;7:623. doi: 10.1038/s42003-024-06314-2 (PMC11130297; doi:10.1038/s42003-024-06314-2)
Supplement: Supplementary file 4 — Reporting summary [file 42003_2024_6314_MOESM4_ESM.pdf]

nature portfolio

Corresponding author(s): Unniappan

Last updated by author(s): Apr 26, 2024

Reporting Summary

Nature Portfolio wishes to improve the reproducibility of the work that we publish. This form provides structure for consistency and transparency in reporting. For further information on Nature Portfolio policies, see our Editorial Policies and the Editorial Policy Checklist.

Statistics

For all statistical analyses, confirm that the following items are present in the figure legend, table legend, main text, or Methods section.

n/a	Confirmed	
☐	☒	The exact sample size (*n*) for each experimental group/condition, given as a discrete number and unit of measurement
☒	☐	A statement on whether measurements were taken from distinct samples or whether the same sample was measured repeatedly
☐	☒	The statistical test(s) used AND whether they are one- or two-sided<br>*Only common tests should be described solely by name; describe more complex techniques in the Methods section.*
☐	☒	A description of all covariates tested
☐	☒	A description of any assumptions or corrections, such as tests of normality and adjustment for multiple comparisons
☐	☒	A full description of the statistical parameters including central tendency (e.g. means) or other basic estimates (e.g. regression coefficient) AND variation (e.g. standard deviation) or associated estimates of uncertainty (e.g. confidence intervals)
☐	☒	For null hypothesis testing, the test statistic (e.g. F, t, r) with confidence intervals, effect sizes, degrees of freedom and P value noted<br>*Give P values as exact values whenever suitable.*
☒	☐	For Bayesian analysis, information on the choice of priors and Markov chain Monte Carlo settings
☒	☐	For hierarchical and complex designs, identification of the appropriate level for tests and full reporting of outcomes
☒	☐	Estimates of effect sizes (e.g. Cohen's *d*, Pearson's *r*), indicating how they were calculated

Our web collection on statistics for biologists contains articles on many of the points above.

Software and code

Policy information about availability of computer code

Data collection	*Provide a description of all commercial, open source and custom code used to collect the data in this study, specifying the version used OR state that no software was used.*
Data analysis	*Provide a description of all commercial, open source and custom code used to analyse the data in this study, specifying the version used OR state that no software was used.*

For manuscripts utilizing custom algorithms or software that are central to the research but not yet described in published literature, software must be made available to editors and reviewers. We strongly encourage code deposition in a community repository (e.g. GitHub). See the Nature Portfolio guidelines for submitting code & software for further information.

Data

Policy information about availability of data

All manuscripts must include a data availability statement. This statement should provide the following information, where applicable:

- Accession codes, unique identifiers, or web links for publicly available datasets
- A description of any restrictions on data availability
- For clinical datasets or third party data, please ensure that the statement adheres to our policy

Data provided as a supplementary document, and are also available upon request from the corresponding author.

Human research participants

Policy information about studies involving human research participants and Sex and Gender in Research.

Reporting on sex and gender	Both male and female mice were used in this research.
Population characteristics	*Describe the covariate-relevant population characteristics of the human research participants (e.g. age, genotypic information, past and current diagnosis and treatment categories). If you filled out the behavioural & social sciences study design questions and have nothing to add here, write "See above."*
Recruitment	*Describe how participants were recruited. Outline any potential self-selection bias or other biases that may be present and how these are likely to impact results.*
Ethics oversight	Followed CCAC guidelines and ethics approval was obtained from institution research ethics board.

Note that full information on the approval of the study protocol must also be provided in the manuscript.

Field-specific reporting

Please select the one below that is the best fit for your research. If you are not sure, read the appropriate sections before making your selection.

☒ Life sciences ☐ Behavioural & social sciences ☐ Ecological, evolutionary & environmental sciences

For a reference copy of the document with all sections, see nature.com/documents/nr-reporting-summary-flat.pdf

Life sciences study design

All studies must disclose on these points even when the disclosure is negative.

Sample size	All data are represented as mean ± SEM. Results presented are pooled from 3 independent studies with at least triplicates for each treatment. All values are shown as mean ± SEM. Statistical analysis was conducted by SPSS statistical software (IBM SPSS Statistics for Windows, Version 23.0, USA). The normality of data and the presence of outliers were assessed by Shapiro-Wilk's test and drawing box plot, respectively. The homogeneity of variances was analyzed by Leven's test. The single comparison was performed by Student's t-test. Multiple comparisons were done by one-way ANOVA followed by Tukey's multiple comparison test. The three-way ANOVA was performed to evaluate the main effect or combination of three independent variables (diet, sex, gene disruption) on response variable (lipogenic or beta-oxidation gene expression) in mice. The significance level was set at $p < 0.05$. Graphs were plotted by GraphPad Prism (GraphPad Software, Inc., Prism 8 for Windows, Version 8.4.2, USA).
Data exclusions	None
Replication	Repeated 2-3 times.
Randomization	None
Blinding	None

Reporting for specific materials, systems and methods

We require information from authors about some types of materials, experimental systems and methods used in many studies. Here, indicate whether each material, system or method listed is relevant to your study. If you are not sure if a list item applies to your research, read the appropriate section before selecting a response.

Materials & experimental systems

n/a	Involved in the study	
☐	☒	Antibodies
☐	☒	Eukaryotic cell lines
☒	☐	Palaeontology and archaeology
☐	☒	Animals and other organisms
☒	☐	Clinical data
☒	☐	Dual use research of concern

Methods

n/a	Involved in the study	
☒	☐	ChIP-seq
☒	☐	Flow cytometry
☒	☐	MRI-based neuroimaging

Antibodies

Antibodies used	Primary antibodies used in this study were monoclonal mouse anti-beta-actin antibody (1:1000, RRID: AB_528068, cat no. JLA20, Developmental Studies Hybridoma Bank, DSHB, University of Iowa, USA), rabbit anti-mouse NUCB1 (1:250, custom synthesized, cat no. 1312-PAC-02, Pacific Immunology, USA), rabbit anti-mouse NUCB2 (1:250, RRID: AB_2891124, cat no. 1312-PAC-01, Pacific Immunology, USA), polyclonal rabbit anti-phospho (Thr172)-AMPKα antibody (1:1000, RRID: AB_330330, cat no. 2531S, Cell Signalling, USA), polyclonal rabbit anti-AMPKα antibody (1:1000, RRID: AB_330331, cat no. 2532S, Cell Signalling, USA). The secondary antibodies used were goat anti-rabbit IgG (H + L)-HRP conjugate antibody (1:5000, RRID: AB_11125142, cat no. 170-6515, Bio-Rad, USA) and goat anti-mouse IgG (H + L)-HRP conjugate antibody (1: 5000, RRID: AB_11125547, cat no. 170-6516, Bio-Rad, USA).
Validation	Validated in house in previous studies.

Eukaryotic cell lines

Policy information about cell lines and Sex and Gender in Research

Cell line source(s)	Human hepatocellular carcinoma cells (HepG2/C3A cells, Catalog # CRL-1074, ATCC, USA)
Authentication	Authenticated by the vendor
Mycoplasma contamination	All cells were tested negative for mycoplasma
Commonly misidentified lines (See ICLAC register)	Not applicable

Animals and other research organisms

Policy information about studies involving animals; ARRIVE guidelines recommended for reporting animal research, and Sex and Gender in Research

Laboratory animals	C57Bl6 mice
Wild animals	Not Applicable
Reporting on sex	Sex of mice used is reported
Field-collected samples	Not Applicable
Ethics oversight	All Nucb1 KO mice studies followed the guidelines of the Canadian Council for Animal Care and were approved by the University of Saskatchewan Animal Care Committee (2012-0033). University of Saskatchewan Animal Research Ethics Board (under the Canadian Council of Animal Care guidelines).

Note that full information on the approval of the study protocol must also be provided in the manuscript.
